# Cannabinoid overrides triggers of GABAergic plasticity in vestibular circuits and distorts the development of navigation

**DOI:** 10.1016/j.isci.2025.112566

**Published:** 2025-04-30

**Authors:** Wei Shi, Kenneth Lap-Kei Wu, Mengliu Yang, Francisco Paulo De Nogueira Botelho, Oscar Wing-Ho Chua, Hui-Jing Hu, Ka-Pak Ng, Ulysses Tsz-Fung Lam, Kin-Wai Tam, Chun-Wai Ma, Daisy Kwok-Yan Shum, Ying-Shing Chan

**Affiliations:** 1 School of Biomedical Sciences, Li Ka Shing Faculty of Medicine, The University of Hong Kong, Pokfulam, Hong Kong SAR, P.R. China; 2 State Key Laboratory of Brain and Cognitive Sciences, The University of Hong Kong, Pokfulam, Hong Kong SAR, P.R. China; 3 Neuroscience Research Centre, The University of Hong Kong, Pokfulam, Hong Kong SAR, P.R. China; 4 Beijing Advanced Innovation Center for Big Data-based Precision Medicine, School of Engineering and Medicine, Beihang University, Beijing, P.R. China

**Keywords:** Natural sciences, Biological sciences, Neuroscience

## Abstract

Early life exposure to cannabis can result in long-lasting deficits in spatial navigation. We ask if the development of this behavior is subject to early life activity of type I cannabinoid receptor (CB1R) in the vestibular nucleus. In rodents, we found that local exposure to CB1R agonist within the first postnatal week, but not thereafter, led to a decline in the induction efficacy of long-term depression at GABAergic synapses (LTD_GABA_), a key step in the hard-wiring of vestibular circuits. Within this critical period, endocannabinoid-mediated LTD_GABA_ at inhibitory neurons was selectively triggered by cholecystokinin, whereas that at excitatory neurons was by serotonin. Neonatal exposure to cannabinoids extended the phase of high GABAergic synaptic plasticity and overrode the synapse-specific, modulatory mechanism for plasticity. Such treatment delayed the postnatal emergence of vestibular-dependent reflexes and deranged adult navigational behavior. Deficits in higher functions are thus attributable to the maldevelopment of sensory processing circuits resulting from early cannabis exposure.

## Highlights

• Early cannabis exposure impairs graviceptive reflexes via presynaptic CB1R
• CB1R activity in early postnatal stages shapes vestibular GABAergic plasticity
• Disinhibitory and inhibitory sites use distinct triggers for eCB-mediated plasticity
• ECB-mediated LTD_GABA_ alters the development of navigation

## Introduction

Sequential emergence of increasingly complex behavior in development involves nature-defined innate developmental programs subject to nurture resultant from experience-dependent fine-tuning of neural circuits mediated by synaptic plasticity.^1^ The endocannabinoid system (eCB) plays crucial roles in modulating plastic processes that refine neural circuits based on experience.^2^ Of particular importance is the action of the eCB system on the plasticity of gamma-aminobutyric acid (GABA)ergic transmission, which in turn sets the level of neural plasticity in neonatal brain circuits. We asked how aberrations in neonatal GABAergic transmission could lead to circuit dysfunction, manifesting as behavioral deficits in patients with perinatal overexposure to cannabinoids.^3^ Vestibular circuits, which undergo significant refinement in the early postnatal period,^4^^,^^5^^,^^6^ are most susceptible to early cannabinoid exposure.

The role of GABAergic neurons in shaping afferent input processing at second-order vestibular neurons was established by pioneering studies in the frog.^7^^,^^8^ Previously, we showed that GABAergic transmission was required for the functional maturation of vestibular circuits,^4^^,^^5^ even with intact inputs from vestibular sensory input.^6^ In this study, we further demonstrate that type I cannabinoid receptor (CB1R) activity in the early postnatal stage sets the trajectory of synaptic plasticity at GABAergic synapses of vestibular nucleus (VN) circuits.

High synaptic plasticity in GABAergic transmission within the critical period and abrupt decrease of plasticity at the end of the critical period is a common mechanism for the fixation of synaptic characteristics in many sensory systems after a postnatal period of input-dependent refinement.^9^^,^^10^^,^^11^ While it is assumed that a period of heightened plasticity within the critical period allows input-guided fine-tuning of developing circuits,^10^ it is not known whether the temporal control of plasticity in GABAergic transmission is required for circuit maturation. In the absence of sensory input, such as during dark rearing, eCB was required for closure of the extended critical period.^12^ Given omnipresent vestibular input is present in neonate^4^^,^^13^^,^^14^ and neuronal expression of CB1R since birth,^15^ we asked how the activity of the eCB system early in the critical period determines the closure of the critical period.

Vestibular outputs are conveyed via distinct projection pathways segregated by function,^13^^,^^14^ with distinct output dynamics.^16^ This implied existence of distinct signal processing circuits within the VN. Evidence suggests that such differences in the processing of sensory inputs arise not only from differing intrinsic properties of neuronal subtypes,^17^ but also from differences in inhibitory inputs.^7^ On the most basic level, inhibitory synapses can be classified as inhibitory motifs when GABAergic neurons impinge on excitatory neurons, or disinhibitory motifs when both the pre- and postsynaptic neurons are inhibitory.^18^ By separating these two motifs during the recording of GABAergic postsynaptic currents in VGAT-Venus mice,^19^ we revealed type B cholecystokinin receptors (CCK_B_R) and serotonin-2A receptors (5-HT_2A_R) as unique physiological triggers of eCB-mediated plasticity at developing disinhibitory and inhibitory motifs, respectively.

Here, we show that early postnatal exposure to CB1R agonist delayed the maturation trajectory of GABAergic plasticity in VN circuits in rodents, with such animals suffering from long-lasting navigational deficit in adulthood. Although the role of motif-specific tuning of plasticity during the critical period remains to be elucidated, behavioral deficits resulting from overriding these triggers by the non-discriminatory stimulation of eCB receptors revealed its importance in circuit maturation. Results, therefore, highlight the importance of both temporal and motif-specific regulation of LTD_GABA_ toward the formation of functional circuits in the VN.

## Results

### A critical period for endocannabinoid modulation of graviceptive behavior

**Figure 1:**
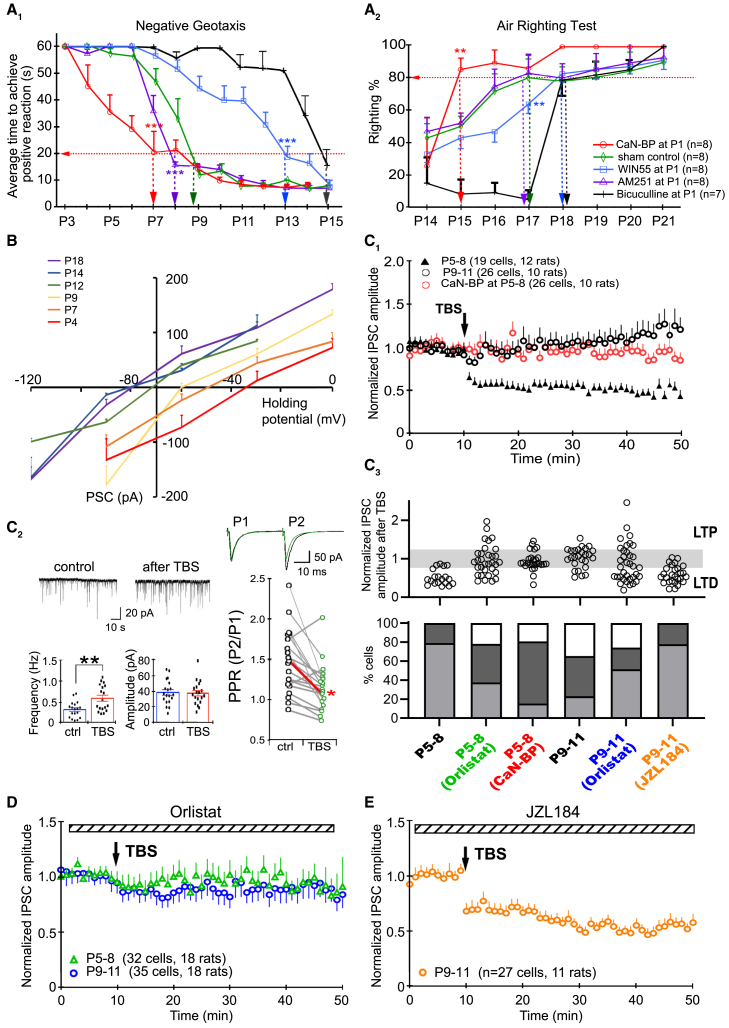
eCB shifts the maturation program of VN circuits through tuning synaptic plasticity at GABAergic synapses (A) The emergence of negative geotaxis (A_1_) and air-righting reflex (A_2_) were delayed with the pretreatment of the VN at P1 with CB1R agonist WIN55 or GABA_A_R antagonist bicuculline, but advanced with CB1R antagonist AM251 or CaN-BP. (B) Gramicidin-perforated whole-cell patch-clamp recording of GABA reversal potential. Postnatal shift of I-V curve indicated that GABAergic response of MVN neurons changed from excitatory to inhibitory between P9 and P12. Vertical axis intersects the horizontal axis at −70 mV (average resting membrane potential). (C_1_) Average response of MVN neurons to TBS in brain slices obtained from P5-8, P9-11 and P5-8 treated with CaN-BP. (C_2_) Following TBS, mPSC_GABA_ frequency was increased (*n* = 19 cells, *p* < 0.001) but amplitude was unchanged (*n* = 19 cells, *p* > 0.05) and PPR was decreased (*n* = 17 cells, *p* < 0.01), indicating presynaptic plasticity. Lines join PPR values from the same cell. Red line indicates change in mean PPR value before and after TBS. Representative tracings of paired-pulse responses before (black) and after TBS (green) are shown above. (C_3_) Top: Normalized PSC amplitudes of each recorded cell after TBS under various experimental conditions. Pale gray area denotes no change in PSC_GABA_ amplitude, the area below for LTD in PSC amplitude, and that above for LTP response. Bottom: Bar chart summarizing the percentage of MVN neurons showing LTD (light gray), LTP (white) or no change in PSC amplitude (dark gray). In P5-8 brain slices, 80% of sampled MVN neurons exhibited TBS-induced LTD_GABA_, while the remaining 20% showed no change. LTP was not observed. With the bath addition of CaN-BP, 65% of sampled MVN neurons showed no response to TBS (red circles in C_1_, *p* < 0.001, vs. untreated P5–8 cells). In P9–11 slices, the occurrence of LTD decreased to 29% while another 32% of sampled MVN neurons exhibited LTP (*p* < 0.001 vs. untreated P5–8 cells). (D) LTD was abolished in the averaged PSC response of all recorded MVN cells in P5–8 brainstem slices after blocking lipase activity with the bath addition of Orlistat. Closer examination of the response of each cell further revealed that the proportion of LTD_GABA_-expressing MVN neurons was significantly decreased to 38% (*p* = 0.008, vs. untreated P5–8 cells, see C_3_). LTP was induced in another 22% of sampled MVN neurons. (E) With the bath addition of JZL184 to P9–11 brainstem slices to inhibit 2-AG degradation, the percentage of MVN neurons that showed LTD_GABA_ increased to 78% (*p* < 0.001 vs. untreated P9–11 cells). Mean ± SEM are shown. ∗*p* < 0.05, ∗∗*p* < 0.01, ∗∗∗*p* < 0.01. two-way ANOVA for behavioral tests in (A), pair t-test for mPSC_GABA_ recordings in (C_2_), Fisher’s exact test with Bonferroni’s correction for multiple measurements was used to evaluate change in responses to TBS in (C_3_).

Localized efflux of CB1R agonist WIN55 above the VN from P1 delayed the emergence of negative geotaxis to P13 (Figure 1A_1_, WIN55-treated vs. sham controls, a blocking peptide against calcineurin^20^^,^^21^^,^^22^ (CaN-BP)- or CB1R antagonist AM251-treated at P13: *p* < 0.001), contrasting with saline controls in which this behavior emerged by P9. The air righting reflex, which normally emerges at P17, was also delayed to P18 by such treatment (Figure 1A_2_, WIN55-treated vs. all other treatments at P17: *p* < 0.01). Contrarily, exposure to AM251 from P1 advanced the emergence of negative geotaxis to P8 (Figure 1A_1_, AM251-treated vs. sham controls, WIN55-, or BIC-treated at P8: *p* < 0.001). The combined treatment of WIN55 and AM251 had no significant effect on either behavioral test (Figure S2). This established that CB1R activity in the early postnatal stage bi-directionally affected the circuit maturation of the medial vestibular nucleus (MVN).

Exposure to WIN55-loaded Elvax from P12 onwards did not affect negative geotaxis (Figure S3B). This time point coincided with the switch in polarity of GABAergic transmission (E_GABA_) in VN from depolarizing to hyperpolarizing at P12 (Figure 1B), a hallmark of the maturation of GABAergic circuits.^23^ We further found that WIN55 exposure at P8, one day before the functional maturation of circuits for negative geotaxis, also caused no delay (Figure S3A). Results corroborate clinical evidence that chronic adult exposure to cannabinoids is largely inconsequential^24^ and highlighted a critical period closing at P8 during which the CB1R directed maturation of VN circuits.

### Decrease in induction efficacy of long-term depression at GABAergic synapses drives circuit maturation

Decreased synaptic plasticity of VN circuits in normal rats at P8, as reflected magnitude of long-term change in synaptic strength after TBS (Figure 1C_1_), accompanied closure of the critical period for vestibular development at this stage.

Examination of individual responses revealed a significant decrease in percentage of MVN neurons displaying an LTD response after TBS, from 80% within the critical period (P5−8) to 20% after the closure of the critical period for VN circuits that support reflexive actions at P9–11 (Figure 1C_3_, *p* < 0.001 vs. P5–8). The magnitude of LTD_GABA_ in cells that continued to show LTD response in P9–11 rats was also reduced compared to those in P5–8 rats (63.46 ± 9.03%, *n* = 6/26 cells, 10 rats, *p* = 0.002) (Figure 1C_3_). Addition of GABA_A_ receptor antagonist bicuculline to the bath after the LTD measurements confirmed that the PSCs observed were indeed mediated by GABA_A_ receptors (Figure S3D). These confirmed that the maturation of MVN circuits and closure of the critical period were also marked by a decrease in synaptic plasticity, as in critical periods of cortical circuits.^25^

We then tested whether decreased LTD_GABA_ incidence could, in turn, promote the functional maturation of VN circuits. Exposure of VN neurons to CaN-BP at P1 decreased LTD_GABA_ incidence in the neonatal VN to 15% (Figure 1C, *p* < 0.001 vs. untreated). Such treatment also advanced the emergence of negative geotaxis from P9 to P7 (Figure 1A_1_), and that of air righting behavior from P17 to P15 (Figure 1A_2_). Contrarily, blockade of GABA_A_ receptors to simulate depressed GABAergic transmission resultant from sustained high LTD_GABA_ incidence, using a bicuculline-loaded Elvax slice, delayed the emergence of negative geotaxis to P15 (Figure 1A_1_) and that of air righting reflex to P18 (Figure 1A_2_). These results suggested that the plasticity of GABAergic transmission is causal for VN circuit maturation.

### Long-term depression at GABAergic synapses in the medial vestibular nucleus during the critical period is mediated by endocannabinoid signaling

To dissect the controlling mechanism for LTD_GABA_ in the VN during the early postnatal period (P5–8), we first determined if pre- or post-synaptic mechanisms were dominant in this period. Significantly increased miniature PSC_GABA_ frequency (0.46 ± 0.03 Hz to 0.64 ± 0.04, *n* = 19 cells, *p* < 0.01) and decreased paired pulse ratio (PPR, 1.49 ± 0.39 to 1.15 ± 0.29, *n* = 19 cells, *p* < 0.001) after TBS (Figure 1C_2_), and unchanging PSC_GABA_ amplitude (before TBS: 38.64 ± 0.82 pA, after TBS: 35.55 ± 0.91 pA, *n* = 19 cells, *p* = 0.874) pointed to the pre-synaptic regulation of GABAergic plasticity.

We therefore tested if the presynaptic CB1R receptor^12^^,^^18^^,^^26^^,^^27^ was a major regulator for synaptic plasticity in the neonatal VN. Blocking synthesis of CB1R ligand 2-arachidonoylglycerol (2-AG) with Orlistat decreased the percentage of P5–8 MVN neurons capable of expressing LTD_GABA_ from 80% (control) to 38% (Figures 1C_3_ and 1D, *p* = 0.008 vs. untreated). Conversely, the inhibition of 2-AG degradation with JZL184 in P9–11 MVN neurons increased the induction efficacy of LTD_GABA_ from 29% (control) to 78% (Figures 1C_3_ and 1E, *p* < 0.001 vs. untreated). It is noteworthy that JZL184 caused a decrease in membrane resistance (Figure S4). These pharmacological manipulations proved that the majority of TBS-induced LTD_GABA_ in the neonatal rat VN was elicited by activity of eCB system.

### Early cannabinoid exposure prolonged high level of long-term depression induction at GABAergic synapses

**Figure 2:**
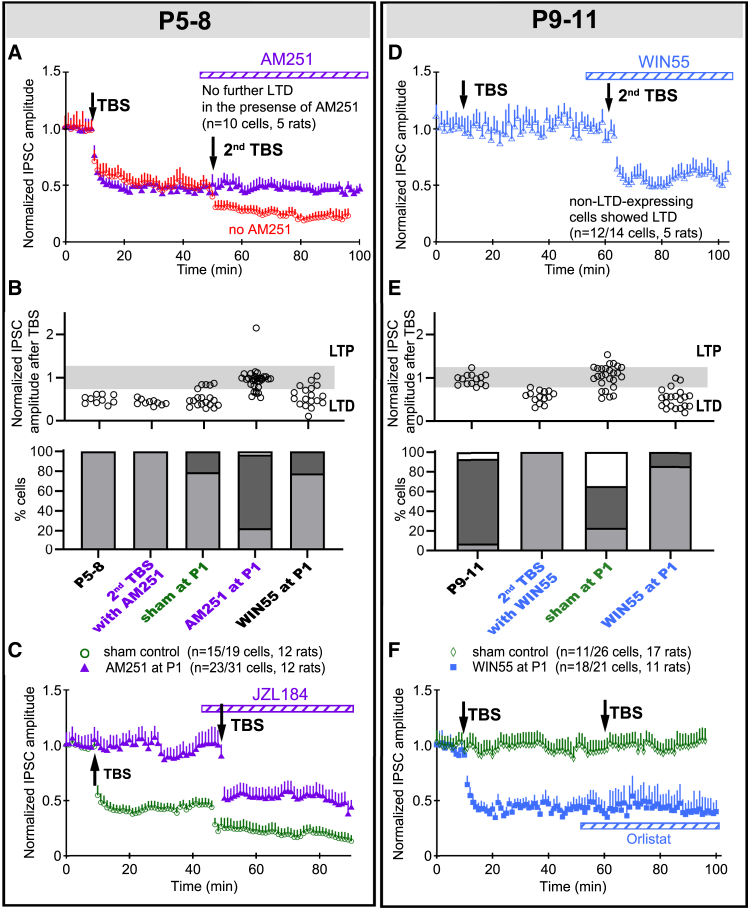
eCB system governs the induction efficacy of LTD_GABA_ and sets the developmental trajectory of LTD_GABA_ in developing VN neurons (A) In P5–8 brain slices, MVN neurons that responded to an initial TBS with LTD_GABA_ could no longer respond to a second TBS with further LTDGABA after the bath addition of AM251 (purple triangles). In a parallel preparation of P5–8 slices (red circles), a second TBS could induce further LTD_GABA_ in LTD_GABA_-expressing MVN neurons. (B) Bar charts summarizing the individual PSC_GABA_ (top) and the percentages (bottom) of P5–8 MVN neurons to TBS after sham or P1 a.m.251 application, as well as the percentage of cells showing each type of response, viz. LTD (light gray), LTP (white) or no change in PSC amplitude (dark gray). (C) In P5–8 rats pre-treated with AM251 at P1, 74% of sampled MVN neurons in brain slices did not exhibit LTD_GABA_ following an initial TBS (purple triangles), contrasting sham control rats in which 79% of sampled neurons showed LTD_GABA_ (green circles, *p* < 0.001). With the bath addition of JZL184, a second TBS could induce LTD_GABA_ in 80% of sampled MVN neurons that were non- LTD_GABA_-expressing with the first TBS (purple triangles). (D) In P9–11 slices, all sampled MVN neurons that did not respond to an initial TBS could then be induced to express LTD_GABA_ with a second TBS after the bath addition of WIN55. (E) Bar charts summarizing the individual PSC_GABA_ (top) and the percentages (bottom) of P9–11 MVN neurons to TBS after sham or P1 WIN55. (F) In P9–11 rats pre-treated with WIN55 (blue squares) at P1, 86% of sampled MV neurons in brain slices exhibited LTD_GABA_ after the first TBS, contrasting sham control rats in which only 23% of sampled MVN neurons showed LTD_GABA_ (*p* < 0.001). These LTD_GABA_-expressing cells could not respond to a second TBS with further LTD_GABA_ after the addition of Orlistat to the bath. Mean ± SEM are shown. Fisher’s exact test Bonferroni’s correction for multiple measurements was used to evaluate change in responses to TBS in (B) and (E).

If so, can CB1R activity set the developmental trajectory of decreasing LTD_GABA_ incidence? Abolishment of further LTD_GABA_ response to a second TBS by the bath addition of AM251 (Figure 2A; Figure S5) or Orlistat (Figure 2F, blue tracing) proved that the observed LTD_GABA_ was indeed mediated by CB1R. In rats pretreated with WIN55 at P1, a high incidence (79%) of eCB-mediated LTD_GABA_ (Figure 2B) was maintained to P9–11 (Figure 2E, 78%), contrasting with 23% in controls (Figure 2E, *p* < 0.001 vs. sham controls). Conversely, pre-treatment with AM251 at P1 decreased the occurrence of eCB-mediated LTD_GABA_ at P5–8 from 79% (sham) to 23% (Figure 2B, *p* < 0.001 vs. sham), indicating the premature suppression of plasticity in MVN circuits. The amplitude of LTD in cells that responded as such to TBS, however, was unchanged by pre-treatment with WIN55 at P1 (46.84 ± 7.83%, *n* = 17/23 cells, 11 rats, *p* = 0.814) or AM251 at P1 (45.42 ± 7.09%, *n* = 8/41 cells, 12 rats, *p* = 0.934) as compared to control rats.

In P9–11 rats, the lower percentage of cells showing LTD_GABA_ after TBS was not due to the developmental downregulation or inactivation of CB1R on these neurons. Using a double TBS protocol, P5–8 or P9–11 MVN neurons which did not display LTD_GABA_ after an initial TBS could be induced to display LTD_GABA_ by increasing synaptic 2-AG concentration with JZL181 (Figure 2C, purple tracing) or direct activation of CB1R with WIN55 (Figure 2D). Additionally, most recorded neurons were excitatory, and WIN55 decreased their excitability (Figure S6). This implied that the retrograde activation of CB1R by postsynaptic release of endocannabinoids was the predominant factor that governed the incidence of LTD_GABA_ in the VN.

### Unique triggers for 2-arachidonoylglycerol release in medial vestibular nucleus circuits

**Figure 3:**
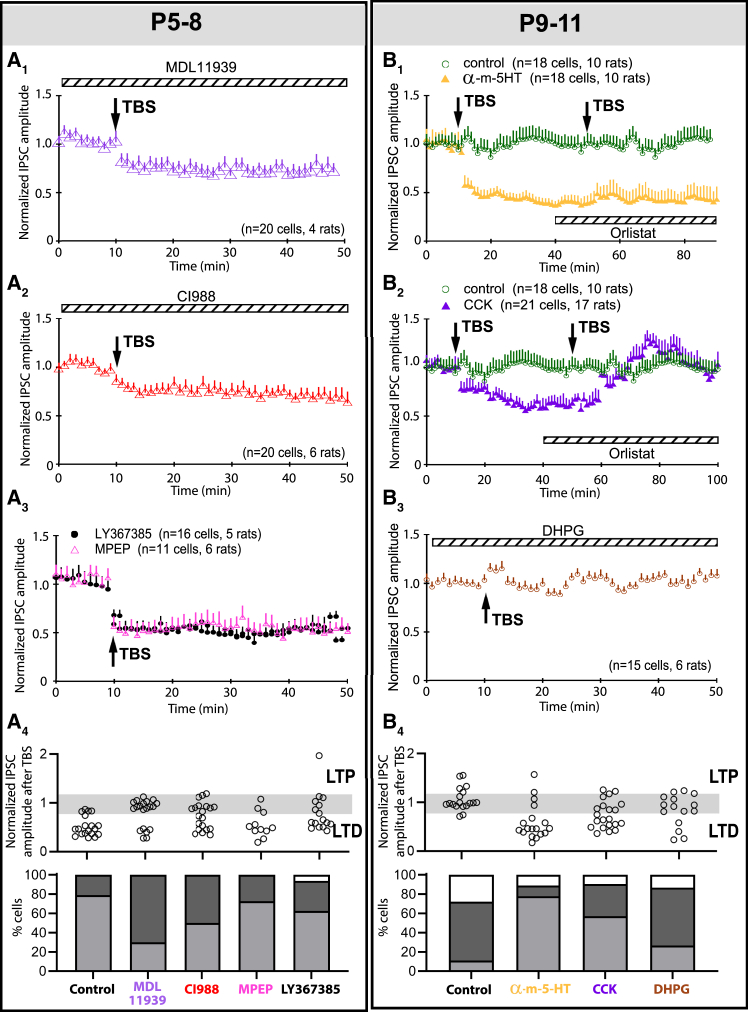
eCB release is triggered by 5-HT and CCK receptors but not metabotropic glutamate receptors in developing VN neurons (A_1_) With the bath addition of MDL11939 (a 5-HT receptor antagonist) to P5-8 brain slices, the percentage of P5–8 MVN neurons showing LTD_GABA_ decreased to 30% (green triangles, *p* = 0.003), contrasting 79% in controls. (A_2_) With the bath addition of CI988 (a CCKBR antagonist) the percentage of P5–8 MVN neurons showing LTD_GABA_ decrease slightly to 50% (CI988, blue triangles), but was not statistically significant (*p* = 0.096). (A_3_) With the bath addition of LY367385 (a mGLuR1 receptor antagonist, black circles) or MPEP (a mGluR5 antagonist, pink triangles), the percentage of P5–8 MVN neurons showing LTD_GABA_ was similar to the control (LY367385: 73%, *p* = 1; MPEP: 62%, *p* = 0.347). (A_4_) Bar charts summarizing the individual PSC_GABA_ (top) and the percentages (bottom) of P5-8 MVN neurons showing each type of response (LTD, light gray; LTP, white; no change, dark gray) in each treatment group after TBS. (B_1_) In P9–11 brain slices, no LTD or LTP was observed in the averaged response of recorded MVN cells (green circles). This was in agreement with the majority (61%) of cells showing no change after TBS (see B_4_). With the bath addition of α-m-5-HT (a 5-HT receptor agonist), the percentage of MVN neurons showing LTD_GABA_ after an initial TBS increased to 78% (yellow triangles, *p* < 0.001). These LTD_GABA_-expressing cells could not respond to a second TBS with further LTD_GABA_ with the addition of Orlistat to the bath. (B_2_) With the bath addition of CCK (a CCKBR agonist), 57% of the P9–11 MVN neurons showed LTD_GABA_ (purple triangles, *p* = 0.011), contrasting control rats in which only 11% of sampled MVN neurons showed LTD_GABA_. (B_3_) With the bath addition of DHPG (a mGluR1 agonist), the percentage of MVN neurons showing LTD_GABA_ after TBS did not change significantly (27%, blue circles, *p* = 0.484). (B_4_) Bar charts summarizing the individual PSC_GABA_ (top) and the percentages (bottom) of P9–11 MVN neurons in each treatment group after TBS. Mean ± SEM are shown. Fisher’s exact test with Bonferroni’s correction for multiple measurements was used to evaluate change in responses to TBS in (A_4_) and (B_4_).

We then tested the efficacy of other G_q_-coupled receptors, such as 5-HT_2A_R and CCK_B_R,^28^^,^^29^ that were less commonly associated with 2-AG release in the forebrain.^30^^,^^31^ Bath application of 5-HT_2A_ receptor antagonist MDL11939 (Figure 3A_1_) significantly decreased the probability of LTD_GABA_ incidence among P5–8 MVN neurons to 30% (*p* = 0.004), while CCK_B_R antagonist CI988 (Figure 3A_2_) produced a slight decrease to 50% (*p* = 0.096) (Figure 3A_4_). While the same canonical PLC-, PKA-, and Ca^2+^-dependent intracellular pathways as those in forebrain neurons^32^^,^^33^ were utilized by MVN neurons to effect eCB mediated LTD_GABA_ (Figures S7A–S7C), MVN neurons utilized distinct transmembrane signaling receptors to trigger 2-AG release. In the forebrain, 2-AG release triggered by metabotropic glutamate receptor (mGluR) is well documented.^34^^,^^35^^,^^36^ Bath application of mGluR5 antagonist MPEP or mGluR1 antagonist LY367385 to MVN slices of P5–8 rats (Figure 3A_3_), however, did not change the induction efficacy of eCB-mediated LTD_GABA_ (Figure 3A_4_, MPEP, *p* = 1; LY367385, *p* = 0.347 vs. control).

Whereas in P9–11 rats, the application of 5-HT_2_R agonist α-m-5-HT (Figure 3B_1_) significantly increased the proportion of cells responding to TBS with LTD_GABA_ to 78% (*p* < 0.001 vs. control), while CCK (Figure 3B_2_) increased the proportion to 57% (*p* = 0.011 vs. control) (summarized in Figure 3B_4_). Such an increase in the proportion of cells showing LTD_GABA_ response was confirmed to be eCB-mediated by the addition of orlistat to the bath which blocked further induction of LTD_GABA_ by a second TBS in P9–11 rat VN neurons despite the presence of CCK (Figure 3B_2_) or α-m-5-HT (Figure 3B_1_). The apparent redundancy of 5-HT_2A_R and CCK_B_R on triggering eCB-mediated LTD_GABA_ in the MVN suggested the possibility of yet unknown mechanisms for differential control of plasticity in GABAergic transmissions. Addition of mGluR1 agonist DHPG (Figure 3B_3_) also did not affect the induction of eCB-mediated LTD_GABA_ in P9–11 cells (Figure 3B_4_, *p* = 0.484 vs. control). These results thus demonstrate that the activation of mGluR1/5 did not trigger postsynaptic release of 2-AG as a retrograde messenger necessary for the induction of eCB-mediated LTD_GABA_ in MVN, distinct from glutamate-dependent triggering eCB-mediated LTD_GABA_ in the hippocampus.^37^

### Cell-type specific control of long-term depression at GABAergic synapses by the differential triggering of endocannabinoid release

**Figure 4:**
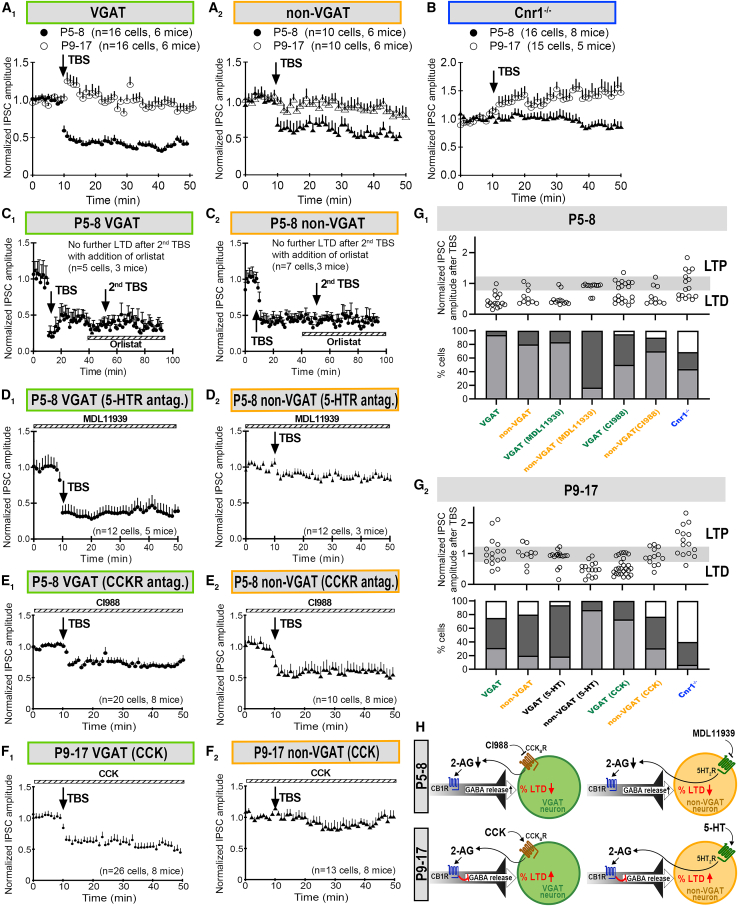
Distinct triggers for eCB-mediated LTD_GABA_ at inhibitory and excitatory neurons in VN (A) In P5–8 VGAT mice (filled circles), the majority of sampled VGAT (A_1_, 40%) and non-VGAT neurons (A_2_, 80%) in the MVN showed LTD_GABA_ after TBS. While at P9–17 (open circles), the majority of neurons showing no change in response amplitude after TBS (A_2_). (B) Cnr1^−/−^ mice had higher percentage of LTP-expressing MVN neurons at both P5–8 (filled triangles, *p* = 0.001), but the difference was no longer significant at P9–17 (open circles, *p* = 0.067) compared with VGAT-Venus mice. (C) In P5–8 mice, LTD_GABA_-expressing neurons in both VGAT (C_1_) and non-VGAT (C_2_) populations could not be induced to express LTD_GABA_ with a second TBS after the bath addition of Orlistat. (D) Bath addition of MDL11939 (a 5HT_2A_R antagonist) to P5–8 brain slices did not affect LTD_GABA_ in VGAT neurons in the MVN (*p* = 0.560) (D_1_). However, the percentage of LTD-expressing non-VGAT cells was significantly decreased to 17% (*p* = 0.008) after the bath addition of MDL11939 (D_2_). (E_1_) With the bath addition of CI988 (a CCK_B_R antagonist) to P5–8 slices, the percentage of LTD_GABA_-expressing VGAT neurons in the MVN decreased to 50% (*p* = 0.009 vs. P5–8 control). (E_2_) Such treatment did not significantly reduce the percentage of LTD_GABA_-expressing non-VGAT neurons in the MVN (70%, *p* = 0.293). (F_1_) With the bath addition of CCK to P9–17 slices, the percentage of LTD_GABA_-expressing VGAT neurons in the MVN was increased to 73% (*p* = 0.004 vs. P9–17 control). (F_2_) On the other hand, the percentage of non-VGAT neurons responding to TBS with LTD_GABA_ remained similar to controls at 31% (*p* = 0.857). (G_1,2_) Bar charts summarizing PSC_GABA_ amplitudes of each recorded cell and percentages of MVN neurons showing the response described above for P5–8 (F_1_) and P9–17 (F_2_) VGAT-Venus and Cnr1^−/−^ mice. (H) Schematic diagram showing differential control of eCB-mediated LTD_GABA_ by CCK_B_R and 5HT_2A_R at VGAT and non-VGAT neurons in the P5–8 and P9–17 MVN. Mean ± SEM are shown. Fischer’s exact test with Bonferroni’s correction for multiple measurements was used to compared response profiles to TBS between various treatment groups.

GABAergic neurons impinging upon excitatory or inhibitory neurons form inhibitory and disinhibitory motifs, respectively.^18^ Being the building blocks of functional circuits,^18^ we reasoned that the tuning of plasticity at such motifs would require distinct regulation. Use of vesicular GABA transporter (VGAT)-Venus transgenic mice^19^ allowed the distinction of plasticity at disinhibitory (Figure 4A_1_) and inhibitory motifs (Figure 4A_2_). The overall profile of TBS-induced neuronal plasticity in VGAT-Venus mice was not different from rats (P5–8 VGAT mice vs. P5–8 rat, *p* = 0.682; P9–17 VGAT-Venus mice vs. P9–11 rat, *p* = 0.694) nor from wild type mice (P5–8, *p* = 0.402; P9–17, *p* = 1).

Moreover, VGAT and non-VGAT neurons had similar incidence of LTD_GABA_ (P5–8, *p* = 0.538; P9–17, *p* = 0.873, Figures 4A_1,2,_ and 4G_1,2_), which were reduced with age (VGAT neurons, *p* < 0.001; non-VGAT neurons *p* = 0.033). The amplitude of LTD_GABA_ after TBS was also similar between VGAT-Venus and rats (P5–8, *p* = 0.779; P9–17, *p* = 0.580). Addition of Orlistat could abolish further induction of LTD_GABA_ in both VGAT (Figure 4C_1_) and non-VGAT cells (Figure 4C_2_) in P5–8 MVN VGAT-mice. These confirm the validity of VGAT-Venus mice as a model for studying LTD_GABA_ in the MVN during early postnatal development.

We revealed the differential modulation of LTD_GABA_ incidence at VGAT versus non-VGAT neurons by the bath addition of 5-HT_2A_R or CCK_B_R agonists/antagonists. In P5–8 MVN slices, the bath addition of 5-HT_2A_R antagonist MDL11939 did not change the induction efficacy of LTD_GABA_ in VGAT neurons compared to untreated controls (Figures 4D_1_ and 4F_1_, *p* = 0.560), but such treatment reduced LTD_GABA_ incidence to 17% in non-VGAT neurons (Figure 4D_2_, *p* = 0.008). While the effect of CI988 was not significant in random patch-clamp recordings of MVN neurons (cf. Figure 4A_2_), cell type-specific recording in P5–8 VGAT-Venus mice revealed that CCK_B_R antagonist CI988 significantly decreased the probability of LTD_GABA_ induction from 94% to 50% in VGAT neurons (Figures 4E_1_ and 4G_1_, *p* = 0.009), but not in non-VGAT neurons (Figures 4E_2_ and 4G_1_, *p* = 0.294). Additionally, the combined bath application of MDL11939 and CI988 did not suppress LTD_GABA_ induction more effectively than either antagonist alone (Figure S8).

Distinct triggering of eCB-mediated plasticity at inhibitory and disinhibitory motifs persisted after closure of the critical period. In P9–17 MVN, 5-HT remained as a trigger for LTD_GABA_ in non-VGAT neurons. Increasing the proportion of LTD_GABA_ responses from 20% to 87% (*p* = 0.001, Figure 4F_2_) but not in VGAT neurons (from 20% to 19%, *p* = 0.227, Figure 4F_2_). On the other hand, the bath addition of CCK continued to increase the proportion of cells responding to TBS with LTD_GABA_ in VGAT neurons (from 31% to 73%, *p* = 0.004, Figures 4F_1_ and 4G_2_) but not non-VGAT neurons (from 20% to 31%, *p* = 0.857, Figures 4F_2_ and 4G_2_). In all, results revealed that the triggering of LTD_GABA_ in non-VGAT neurons was 5-HT_2A_R-dependent while that in VGAT neurons was CCK_B_R-dependent (Figure 4H), and this phenomenon is calcium-independent (Figure S9).

### Long-term plasticity in cannabinoid receptor 1 knock-out mice

**Figure 5:**
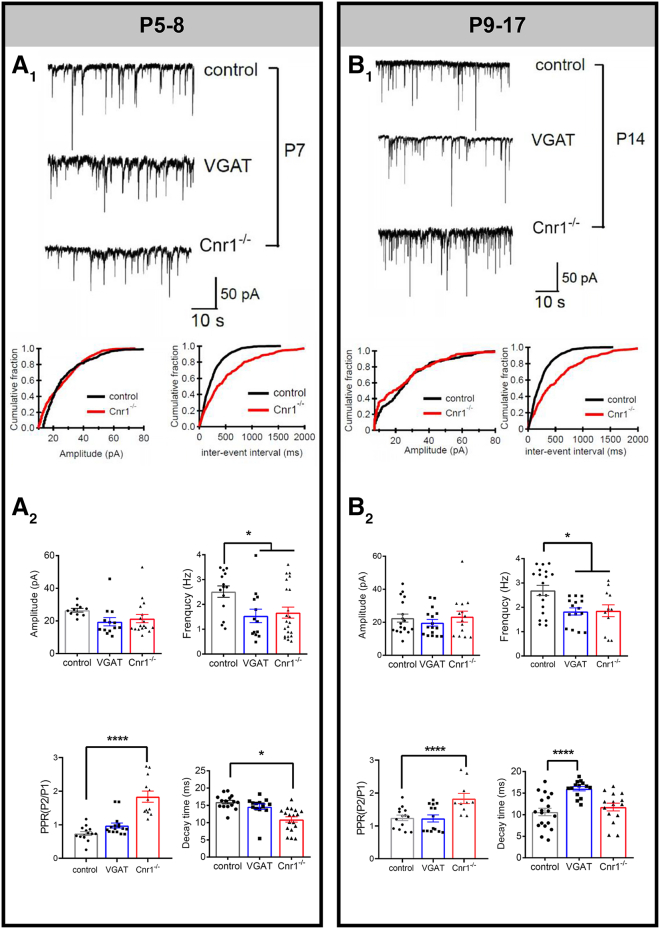
Use of Cnr1^−/−^ to show that GABA release from VN neurons is limited by CB1R. mPSC_GABA_ of MVN cells from control, VGAT and Cnr1^−/−^ transgenic mice of two age groups, viz. P5–18 and P9–17 (A_1_ and B_1_) *Upper panels*: Representative tracings of mPSC_GABA_ from P7 (A_1_) and P14 (B_1_) neurons voltage clamped at −70 mV. *Lower panels*: Cumulative distribution of amplitude and inter-event interval of mPSC_GABA_ from control (black line) and Cnr1^−/−^ mice (red line). (A_2_ and B_2_) The mPSC_GABA_ amplitude of MVN neurons was similar between all groups at both ages, but the mPSC_GABA_ frequency and PPR of MVN neurons was significantly increased in Cnr1^−/−^. This suggested a presynaptic site of action for Cnr1^−/−^. Decay time of mPSC_GABA_ was significantly shorter in Cnr1^−/−^ mice of the P5–8 group but was not different at P9–17 group. Mean ± SEM are shown. ∗*p* < 0.05, ∗∗∗∗*p* < 0.0001. K-S test to compare cumulative distributions in (A_1_) and (B_1_). two-way ANOVA for comparison of mPSC_GABA_ properties in (A_2_) and (B_2_).

The role of eCB system on GABAergic synaptic transmission and plasticity was further confirmed using CB1R knock-out (Cnr1^−/−^) mice. The amplitude of miniature IPSC_GABA_ was similar between control and knockout mice both at P5–8 (Figure 5A_1,2_, *p* = 0.14) and P9–17 (Figure 5B_1,2_, *p* = 0.83). Decay time of mPSC_GABA_ was shorter in P5–8 Cnr1^−/−^ mice compared to controls (Figure 5A_2_, *p* = 0.037). The frequency of mPSC_GABA_ in Cnr1^−/−^ mice was significantly lower than age-matched controls (Figures 5A_2_ and 5B_2_, P5–8: *p* = 0.014, P9–17: *p* = 0.04) with longer inter-event interval (Figures 5A_1_ and 5B_1_, *p* = 0.03) throughout the period investigated (P5–17). This decreased probability of GABA release suggested less depression of GABAergic transmission in Cnr1^−/−^ mice. Moreover, significantly increased PPR in Cnr1^−/−^ mice throughout this period compared to controls (Figures 5A_2_ and 5B_2_, P5–8: *p* < 0.0001, P9–17: *p* < 0.0001) supported the notion of a presynaptic site of action for CB1R.

Whole-cell patch-clamp recording from MVN neurons in Cnr1^−/−^ mice further confirmed the role of CB1R on LTD_GABA_ induction. Incidence of LTD_GABA_ was significantly lower in P5–8 Cnr1^−/−^ mice (44% in Cnr1^−/−^, Figure 4G_1_) compared to VGAT-Venus mice (88%, *p* = 0.001). After the maturation of VN circuits at P9–17, incidence of LTD_GABA_ was no longer different between Cnr1^−/−^ (7%, Figure 4F_2_) and VGAT-Venus mice (27%, *p* = 0.07) mice after the maturation of VN circuits. These results highlight the importance of CB1R in controlling LTD_GABA_ response among developing VN circuits.

### Long-lasting effects of neonatal perturbation of type I cannabinoid receptor in the vestibular nucleus on the maturation of spatial navigation

**Figure 6:**
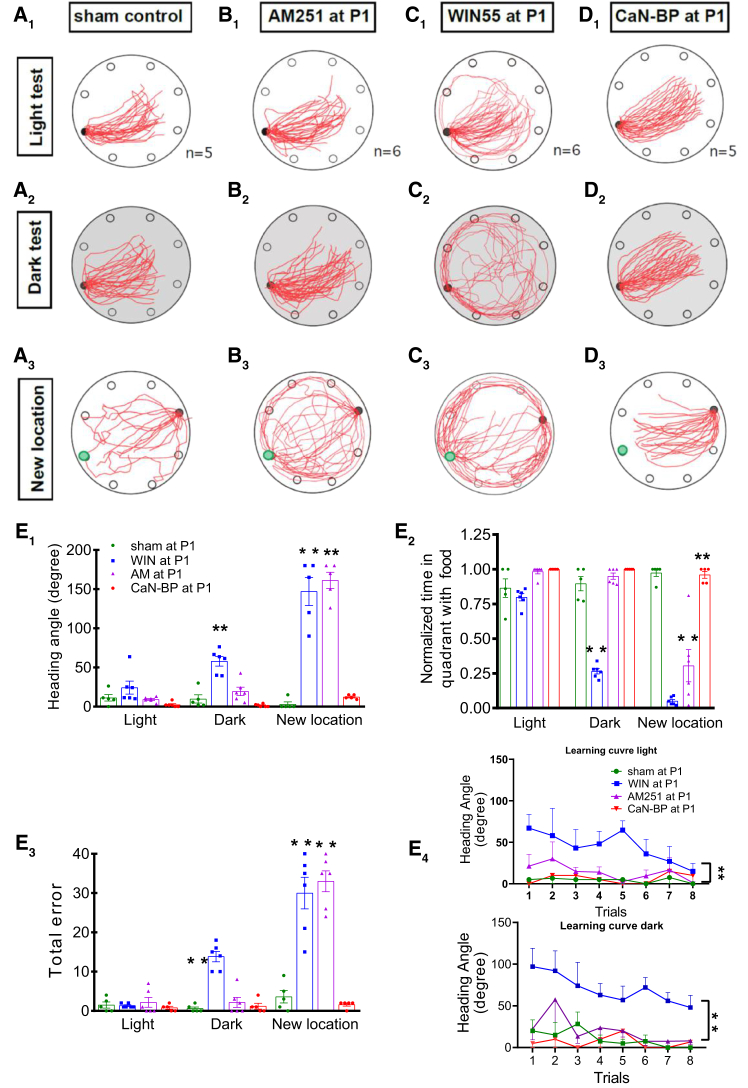
Perturbation of eCB signaling in the neonatal VN leads to long-lasting deficits in vestibular-dependent navigation (A) Excursion paths of adult rats pretreated at P1 with sham implantation, AM251, WIN55, or CaN-BP in the light, dark and new location test for spatial reckoning. Red lines represent the trajectories of homeward paths (light/dark test: 8 trails/rat are superimposed; new location: 4 trails/rat are superimposed). Filled black circle on the edge of each round table surface represents the location of the home base; green circles in bottom panels represent the location of the old home base. Histograms showing the average heading angle (B), time spent in the quadrant containing food (C), errors made in locating the homebase (D), and training time required for rats pretreated with AM251 (*n* = 6 rats), WIN55 (*n* = 6 rats), CaN-BP (*n* = 5 rats), or sham operated (*n* = 5 rats) at P1 or P12 (E). Means ± SEM are shown. ∗*p* < 0.05, ∗∗*p* < 0.01. two-way ANOVA for comparison of behavioral test performance in (B–E).

Direct dosing of CB1R agonist and antagonist bypassed motif-specific regulatory mechanisms for 2-AG release, with corresponding alteration to the duration of the neonatal period of high induction efficacy of LTD_GABA,_ and were accompanied by shifts in the maturation of MVN circuits for reflexes. Given that VN outputs also inform spatial cognition, we reasoned that the perturbed maturation of MVN circuits further impacts multimodal functions such as spatial cognition.^14^ The dead reckoning test was used to assess the spatial cognition of adult rats^38^ (Figure S10). In the dark probe test, where visual signals are absent, vestibular signals become the dominant sensory input for navigational behavior.^38^ Sham control rats acquired the task significantly faster than adult rats pretreated with WIN55 at P1 (Figure 6E, *p* < 0.01). Analysis of the pattern of homeward paths of all WIN55-pretreated rats (Figure 6A, third column from the left), revealed deficits in homeward navigation (i.e., increase in heading angle, time spent in the food quadrant, and error in locating the homebase) in the dark (*p* < 0.01 for all parameters). Normal performance of these rats in the light probe test supported a vestibular origin for the deficit (Figures 6B−6D). Early suppression of LTD_GABA_ induction efficacy with CaN-BP (Figure 1C) or AM251 pretreatment at P1 (Figure 2B) did not cause navigational impairment in either the light or dark probe test (Figures 6A–6D). However, when spatial memory played a more significant role, such as when presented with conflicting visual and vestibular cues in the new location test, both WIN55- and AM251-pretreated rats had difficulty finding their way back to the new home (Figures 6A−6D).

Absence of navigational deficits both in the light and dark probe tests despite perturbation with WIN55 or AM251 at P8 or P12 implied the normal operation of adult circuits for processing visual and vestibular inputs (Figures S11 and S12). This demonstrated that the critical period for vestibular-dependent navigation was also closed by P8.

## Discussion

Retardation of neurodevelopment by early exposure to cannabinoids has been documented^3^ but the mechanism of this has remained unclear. By following the maturation of the vestibular system in the early postnatal stage, this study provides evidence that control of LTD_GABA_ by CB1R is crucial not only for the timely maturation of local reflexive circuits in the MVN but also permanent establishment of vestibular-dependent higher functions such as navigation. We revealed specific triggers for LTD_GABA_ at inhibitory and disinhibitory motifs within the MVN. Bypassing these triggers with the non-discriminatory activation of presynaptic CB1R in the early postnatal period sustained high occurrence of plasticity in GABAergic transmission beyond the normal duration of the critical period. This led to delayed postnatal emergence of vestibular-dependent reflexes and deficits in adult spatial cognitive performance. Taken together, the results provide a mechanistic link between early cannabinoid exposure and neurodevelopmental deficits.

### Bidirectional impact of neonatal endocannabinoid system activity on maturation profile of graviceptive reflexes

GABAergic neurons play a key role in the processing of afferent inputs in VN circuits.^5^^,^^6^^,^^7^^,^^8^ While temporal control of key electrophysiological events, such as excitatory-inhibitory switch of GABAergic transmission in the forebrain^39^ are known to affect maturation, the contribution of temporal control of the plasticity in GABAergic transmission remains unclear. We show that long-term plasticity in GABAergic transmission within the MVN circuits is attenuated by the end of the critical period at P8, similar to other sensory systems.^11^^,^^40^^,^^41^ This, in theory, allows higher levels of depolarizing GABAergic transmission to provide the excitatory drive for the consolidation of neuronal circuits,^23^^,^^42^^,^^43^ as well as prevent further activity-dependent pruning of synapses.

CB1R activity is known to be subject to homeostatic regulation, including decreased receptor expression^44^ and phosphorylation of the cytoplasmic tail of the receptor upon repeated dosing of WIN55 over a few days.^45^^,^^46^ However, such desensitization is dependent on both age and brain location, with cerebellar neurons of adolescent rats showing non-significant levels of desensitization after prolonged cannabinoid dosing.^47^ While desensitization was not investigated in this study, the desensitization of CB1R in MVN after WIN55 dosing, if present, would further highlight the sensitivity of early postnatal M VN circuit to transient perturbations in CB1R activity.

In addition, we have observed that P9-17 Cnr1^−/−^ mice expressed LTP in MVN neurons (Figure 4B; Figure S13). This suggests that normal CB1R activity is critical for the timely regulation of LTD_GABA_ and, by extension, for the proper development of vestibular circuits. Furthermore, the absence of CB1R disrupts the balance between LTP and LTD, which normally helps to refine synaptic connections during development.^1^ LTP in these mice might result from an unregulated, excessive excitatory drive or from compensatory mechanisms due to the loss of CB1R-mediated inhibition. This unregulated plasticity could disrupt normal circuit development, potentially mirroring or exaggerating the effects seen with early cannabinoid exposure.

Interestingly, decay time of mPSC_GABA_ was shorter in P5–8 Cnr1^−/−^ mice compared to controls (Figure 5A). Presynaptic CB1 receptors influence postsynaptic response as evidence show that endocannabinoids selectively inhibit a subclass of synapses distinguished by their fast kinetics and large unitary conductance.^48^ Cnr1^−/−^ mice which lack CB1 receptors might lead to compensatory changes in the expression or subtype composition of GABA receptors. There might be an increase in the expression of GABA_A_ receptor subtypes that have faster desensitization kinetics, which could contribute to the shorter decay time.

### 5-HT_2A_R and CCK_B_R are involved in the retrograde regulation of long-term depression induction in vestibular nucleus

Having established a role for eCB in modulating the development of early postnatal MVN, we further asked how eCB release itself is regulated to suit circuit-specific needs. MVN neurons utilize 5-HT_2A_ and CCK_B_ receptors to trigger activity-dependent release of 2-AG instead of the more common mGluR. These dual triggers allowed LTD_GABA_ at disinhibitory motifs to be modulated specifically by CCK_B_R, whereas that at inhibitory motifs by 5-HT_2A_R. Thus far, inhibitory dynamics have only been described for second order VN neurons in the mature animal,^7^^,^^8^^,^^49^ with little known about the function and maturation processes of disinhibitory motifs within the VN. Due to non-specific depolarization resultant from bipolar electrode stimulus and the placement of electrodes at the medial margin of the MVN,^50^^,^^51^ GABA-on-GABA inputs to the VN such as those from commissural inhibition,^49^ ipsilateral GABAergic neurons outside the VN similar to those for glycinergic inputs,^52^ or local VN interneurons could not be differentiated. Nonetheless, results revealed a layer of complexity for plasticity in the processing of vestibular information beyond previously reported synaptic plasticity in excitatory inputs to GABAergic versus non-GABAergic VN neurons.^53^

In a normal developing system, CCK and 5-HT work in a coordinated manner to regulate LTD_GABA_ at inhibitory and excitatory synapses, respectively. This coordination ensures a balanced development of the vestibular circuits and proper sensory processing. With early cannabinoid exposure, this coordination is disrupted. The failure to synchronize CCK and 5-HT activity properly results in improper circuit formation and associated behavioral deficits.

### Effects of neonatal exposure to AM251 and WIN55 on spatial navigation

The vestibular nucleus, a key part of this system, integrates signals from the inner ear and sends them to various brain regions to help controlling reflexes and movements.^54^ Neurons in the VN involves processes LTD at synapses, which helps fine-tune the responses of vestibular circuits.^55^ As revealed in our findings, early eCBs exposure disrupts this plasticity, leading to the impaired development of navigation, which may manifest as problems with coordination and movement in adulthood. Vestibular cues form the basis for the relevant integration of visual information into higher circuits that guide spatial navigation.^56^^,^^57^^,^^58^

While behavior-dependent recruitment of distinct subpopulations of MVN neurons to various vestibulo-thalamocortical pathways, and more importantly molecular or electrophysiological evidence for distinct critical periods in such subpopulations, remains to be provided. Our observations provided behavioral evidence supporting a later closure date in critical periods of VN neurons that support higher centers involved in more complex multi-modal tasks compared to those supporting reflexive actions. Given real world tasks are more complex than standardized behavioral tests, the sensitivity period toward cannabinoid exposure might extend even longer into the juvenile stage in humans.

Taken together, we show that neonatal exposure to cannabinoid agonists impacted sensorimotor function in rodents, particularly causing spatial cognitive deficits that persisted into adulthood. Our work unveils specific molecular and cellular mechanisms for the eCB-mediated regulation of the induction efficacy of LTD_GABA,_ necessary for the choreographed entrainment of developing MVN circuits. The existence of motif-specific triggers for eCB-mediated plasticity opens avenues toward understanding how eCB modulates network dynamics and behavioral learning. These regulatory mechanisms of plasticity hold the keys to potential therapies that target dysregulated plasticity commonly found in neuropsychiatric disorders.

### Limitations of the study

The origin of GABA-on-GABA inhibitory inputs was not revealed under the current stimulation paradigm. Nonetheless, results revealed a layer of complexity for plasticity in the processing of vestibular information beyond previously reported synaptic plasticity in excitatory inputs to GABAergic versus non-GABAergic VN neurons.

Behavior-dependent recruitment of distinct subpopulations of MVN neurons to various vestibulo-thalamocortical pathways remains unclear. More importantly, molecular or electrophysiological evidence for distinct critical periods in such subpopulations awaits further investigation. Our observations provided behavioral evidence that VN neurons that support higher centers involved in complex multi-modal tasks have a later closure date in critical periods as compared to those supporting reflexive actions. Given that real world tasks are more complex than standardized behavioral tests, we expect the sensitivity period to exogenous cannabinoid exposure to extend into the juvenile stage in humans.

## Resource availability

### Lead contact

Further information and requests for resources should be directed to and will be fulfilled by the lead contact, Ying-Shing Chan (yschan@hku.hk).

### Materials availability

This study did not generate new materials.

### Data and code availability

• Raw data may be obtained from the corresponding author upon reasonable request.
• No code was generated from this work.
• All reagents, chemicals, and behavioral apparatus were either obtained commercially or could be assembled according to the methods using freely available materials.

## Acknowledgments

We thank Professor Yuchio Yanagawa for VGAT-Venus mice; Simon S.M. Chan for constructing the setups for negative geotaxis, air righting reflex, dead reckoning test, and *in vitro* electrophysiological recording; Alice Y.Y. Lui, Tony Xiaotong Liang and Florence Yu Sum Keung for assistance in the preparation of Elvax and behavioral tests; Kimmy F.L. Tsang for assistance with histology and radioisotope work. This work was funded by Beijing Natural Science Foundation (Grant No. Z200024, Z240011 and L222097 to W.S.), Beijing Hospital Authority Clinical Medicine Development Special Fund (ZLRK202333 to W.S.), Hong Kong Research Grants Council (GRF 761812 to D.K.Y.S.and Y.S.C.), 10.13039/501100005847HMRF Grant (06172866 to K.L.K.W. and Y.S.C.), 10.13039/501100024334State Key Laboratory of Brain and Cognitive Sciences, HKU, and the Chi Lin Kok Ng BHL Foundation.

## Author contributions

W.S., K.L.K.W., C.W.M., D.K.Y.S., and Y.S.C. designed the study. W.S., M.Y., F.P.B., and H.J.H. performed electrophysiological recordings. W.S., K.L.K.W., O.W.H.C., H.J.H., K.P.N., U.T.F.L., and K.W.T. performed behavioral experiments. W.S. and O.W.H.C. conducted immunohistological experiments. K.L.K.W., C.W.M., O.W.H.C., K.W.T., and Y.S.C. designed, made, and implanted the Elvax slices. W.S., M.Y., F.P.B., and Y.S.C. analyzed the electrophysiological data. W.S., K.L.K.W., C.W.M., D.K.Y.S., and Y.S.C. analyzed the behavioral data. W.S. wrote the first draft of the article. K.L.K.W, D.K.Y.S., and Y.S.C. wrote and edited the article. D.K.Y.S., and Y.S.C. conceptualized and supervised the study. W.S., K.L.K.W, D.K.Y.S., and Y.S.C. were responsible for securing funding for this study.

## Declaration of interests

The authors declare that they have no competing interests.

## STAR★Methods

### Key resources table

**Table:** 

REAGENT or RESOURCE	SOURCE	IDENTIFIER
**Chemicals, peptides, and recombinant proteins**		

WIN55 ((3R)-2,3-dihydro-5-methyl-3-(4-morpholinylmethyl)pyrrolo [1,2,3-de]-1,4-benzoxazin-6-yl)-1-naphthalenyl-methanone, monomethanesulfonate)	Tocris Bioscience 1038/10	
CNQX	Tocris Bioscience 0190/10	
D-AP5	Tocris Bioscience 0106/1	
FK506	Tocris Bioscience 3631	
BIC (bicuculline methiodide)	Tocris Bioscience 0131/10	
TTX	Sigma-Aldrich 554412	
AM251 (N-(Piperidin-1-yl)-5-(4-iodophenyl)-1-(2,4-dichlorophenyl)-4-methyl-1H-pyrazole-3-carboxamide)	Tocris Bioscience 1117/1	
SR141716A (N-(Piperidin-1-yl)-5-(4-chlorophenyl)-1-(2,4-dichlorophenyl)-4-methyl-1H-pyrazole-3-carboxamide hydrochloride)	Tocris Bioscience 0923/10	
BAPTA AM	Tocris Bioscience 2787/25	
7,8-dihydroxyflavone (7,8-dihydroxy-2-phenyl-4H-1-benzopyran-4-one)	Tocris Bioscience 3826/10	
MPEP (2-methyl-6-(phenylethynyl) pyridine hydrochloride)	Tocris Bioscience 1212/10	
K252a ((9S,10R,12R)-2,3,9,10,11,12-hexahydro-10-hydroxy-9-methyl-1-oxo-9,12-epoxy-1H-diindolo[1,2,3-fg:3′,2',1′-kl]pyrrolo[3,4-i][1,6]benzodiazocine-10-carboxylic acid methyl ester)	Tocris Bioscience 1683/200U	
LY367385 ((S)-(+)-α-Amino-4-carboxy-2-methylbenzeneacetic acid), DHPG (RS)-3,5-dihydroxyphenylglycine	Tocris Bioscience 1237/10	
JZL184 (4-[Bis(1,3-benzodioxol-5-yl)hydroxymethyl]-1-piperidinecarboxylic acid 4-nitrophenyl ester)	Tocris Bioscience 3836/10	
Orlistat (N-formyl-L-leucine (1S)-1-[[(2S,3S)-3-hexyl-4-oxo-2-oxetanyl]methyl]dodecyl ester)	Tocris Bioscience 3540/10	
CCK (cholecystokinin)	Tocris Bioscience 1166/1	
CI988	Tocris Bioscience 2607/10	
U73122 (1-[6-[[(17β)-3-Methoxyestra-1,3,5(10)-trien-17-yl]amino]hexyl]-1*H*-pyrrole-2,5-dione)	Tocris Bioscience 1268/10	
KT5720 ((9*R*,10*S*,12*S*)-2,3,9,10,11,12-Hexahydro-10-hydroxy-9-methyl-1-oxo-9,12-epoxy-1*H*-diindolo[1,2,3-*fg*:3′,2',1′-*kl*]pyrrolo[3,4-*i*][1,6]benzodiazocine-10-carboxylic acid, hexyl ester)	Tocris Bioscience 1288/100U	
MDL11939 (α-Phenyl-1-(2-phenylethyl)-4-piperidinemethanol)	Tocris Bioscience 0870/10	
α-m-5HT (α-Methyl-5-hydroxytryptamine maleate)	Tocris Bioscience	

### Experimental model and study participant details

#### Animals

Sprague-Dawley rats (Charles River Lab), C57Bl6/J mice (Jackson Laboratory), vesicular GABA transporter (VGAT)-Venus transgenic mice^19^ (a gift from Professor Y Yanagawa, Gunma University Graduate School of Medicine), and CB1R knockout (Cnr1^−/−^) mice (Shanghai Model Organisms Center Incorporated) were used. For experiments conducted on adults, only male rats were used. Early postnatal animals were randomly picked for electrophysiological and behavioral experiments as the sex of rodents prior to weaning is not explicit. Procedures were approved either by The University of Hong Kong Committee on the Use of Live Animals in Teaching and Research or by Beihang University Ethics Review Board.

### Method details

#### Surgery implantation of Elvax slice

200 μL of 10 mM CB1 receptor agonist WIN55 and/or 10 mM CB1 receptor antagonist AM251 solution in dimethyl sulfoxide (DMSO, 4%, Sigma) were mixed and snap frozen with a 10% (w/v) Elvax solution in dichloromethane as described previously. Solidified slices were kept at −20 C to allow evaporation of dichloromethane. Slices cut to final dimensions (1 mm × 1 mm, 200 μm thickness) prior to implantation to the 4^th^ ventricle via the foramen magnum.

#### Dead reckoning behavioral test

Dead reckoning test for spatial cognition was conduction on P60 rats implanted with drug-loaded Elvax slices at P1, P8 or P12, and sham operated rats. Rats were fasted for 12 h prior to test sessions. Only one food pellet (1 g, Supreme Mini-Treats, Bio-Serv) was provided during each test trial. Rats foraged for the food pellet placed randomly around the middle of the circular arena before returning to their home cage in 1 of the 8 possible locations around the arena. Stationary visual cues were provided during the training sessions and light probe tests. In the dark probe test, the lights were switched off and the arena was surrounded completely by a ceiling-to-floor black curtain. The new home location test was conducted in light with the home base moved to the hole diametrically opposite to its original home base. Eight trials were done in each of the light or dark probe tests and 4 in the new location test spread over 3 consecutive days (Figure S10). Heading angle, time in the quadrant with food, errors the rats made in return path, and the training time needed before rats learnt the task were measured from recorded video footage.

#### Patch clamp recording

Borosilicate glass pipettes (4–6 MΩ) filled with internal solution for voltage clamp containing (in mM): 140 KCl, 2 MgCl_2_, 2 Na_2_ATP, 1 ethylene glycol-bis (b-aminoethyl ether)-N,N,N′,N′-tetra-acetic acid (EGTA), and 10 N-2-hydroxyethylpiperazine-N′-2-ethanesulphonic acid (HEPES) (adjusted to pH 7.3, 285–295 mOsm) were used. KCl-based internal solution was used to record evoked GABAergic postsynaptic currents (ePSC_GABA_). For current-clamp recordings, electrodes were filled with an internal solution containing the following (in mM): 134 K-gluconate, 6 KCl, 10 HEPES, 4 NaCl, 7 K2-phosphocreatine, 0.3 NaGTP, and 4 Mg-ATP (pH 7.3 adjusted with KOH). No series resistance compensation was applied but the cell was discarded if the access resistance changed significantly (>25%) during the course of recording. Cell recording was discarded if the leaking current was >100 pA.

### Quantification and statistical analysis

The statistical analyses were performed using GraphPad Prism 9 software. All statistical details of the experiments can be found in the figures and figure legends. All data are presented as mean ± SEM. One-way ANOVA followed by Bonferroni’s correction for multiple comparison was used to compare the average time for accomplishing positive responses in negative geotaxis and air righting tests, as well as performance indexes in the dead reckoning test. Differences with *p* < 0.05 were considered statistically significant.
