# Supplementary material for: Cannabinoid overrides triggers of GABAergic plasticity in vestibular circuits and distorts the development of navigation
Source: iScience. 2025 Apr 30;28(6):112566. doi: 10.1016/j.isci.2025.112566 (PMC12150048; doi:10.1016/j.isci.2025.112566)
Supplement: Document S1. Figures S1–S13 and Methods S1 [file mmc1.pdf]

iScience, Volume *28*

Supplemental information

Cannabinoid overrides triggers of GABAergic plasticity in vestibular circuits and distorts the development of navigation

Wei Shi, Kenneth Lap-Kei Wu, Mengliu Yang, Francisco Paulo De Nogueira Botelho, Oscar Wing-Ho Chua, Hui-Jing Hu, Ka-Pak Ng, Ulysses Tsz-Fung Lam, Kin-Wai Tam, Chun-Wai Ma, Daisy Kwok-Yan Shum, and Ying-Shing Chan

This section includes Supplementary Figures S1-S13 with respective legends, and Supplementary Methods.

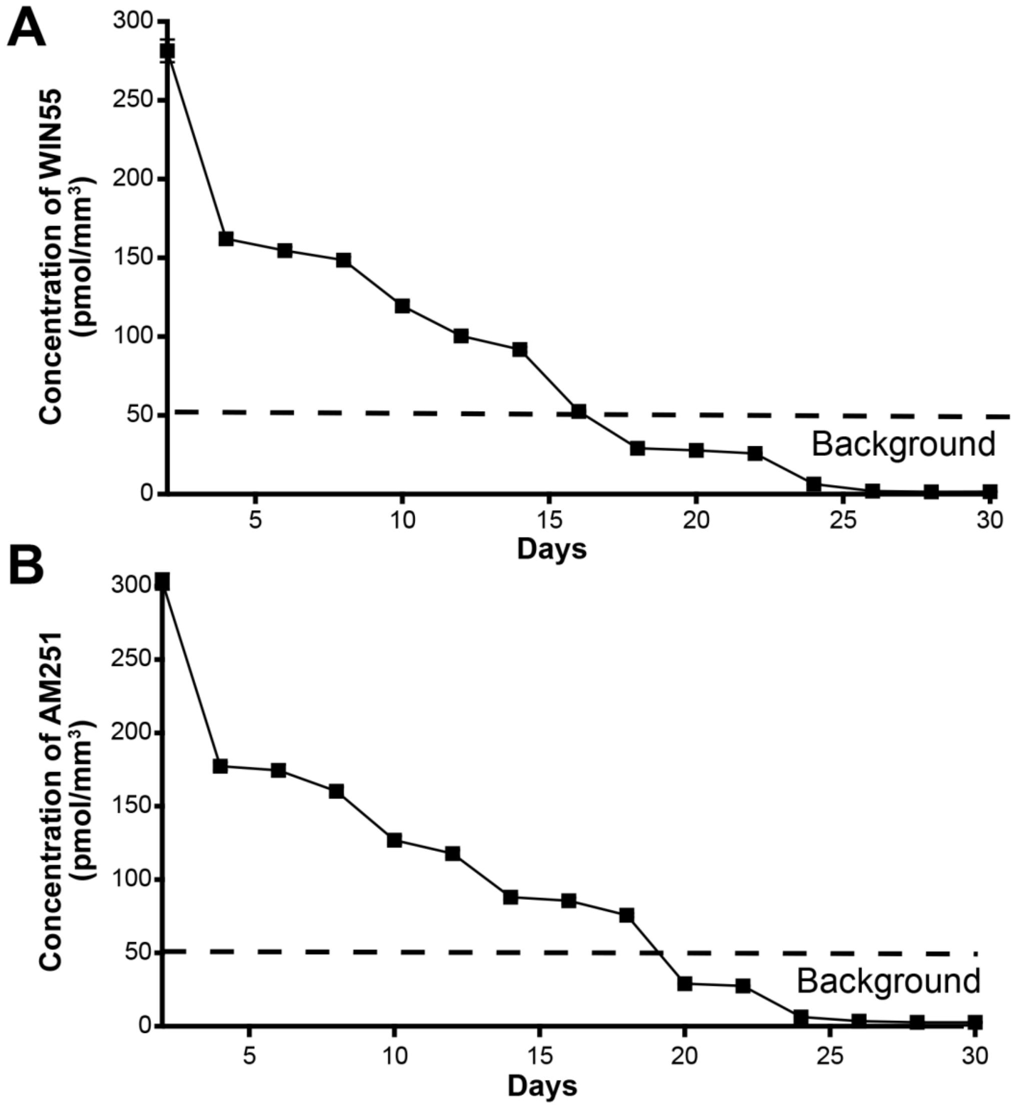

Fig. S1. Characteristics of WIN55 or AM251 effusion from Elvax slice (200 μm thickness), related to Figure 1. (A) Graph showing the amount of drug effused from Elvax slice loaded with 10 mM ^{3}H-WIN55. (B) Graph showing the amount of drug effused from Elvax slice loaded with 10 mM ^{3}H-AM251.

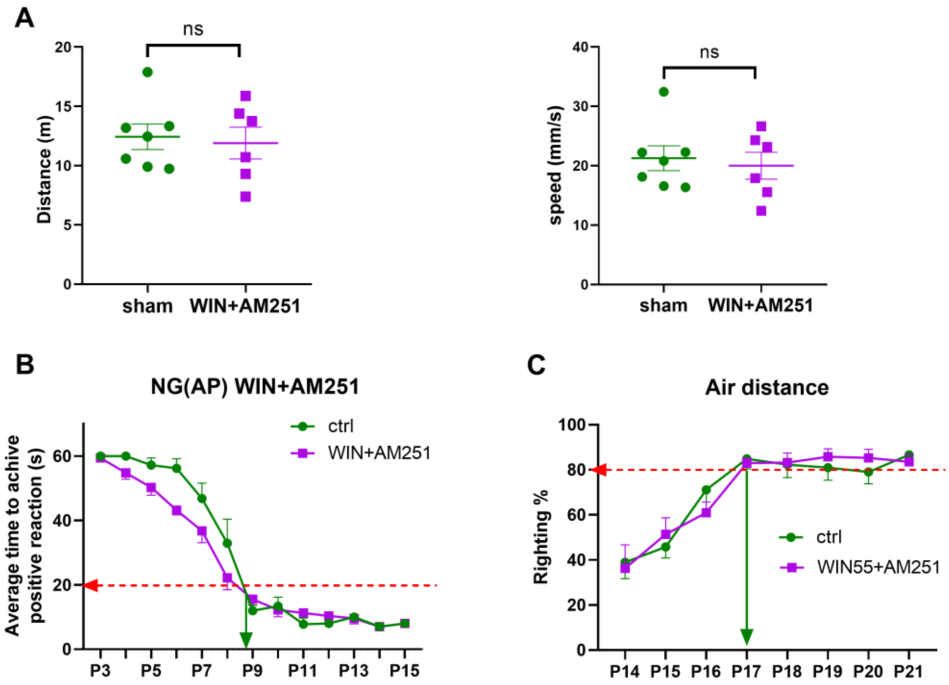

Fig S2. Combined treatment of WIN55 and AM251, related to Figure 1. (A) open field tests for P21 rats on sham control group at P1 group and WIN+AM251 pretreated at P1 group, which show no significant different between two groups. (B, C) The emergence of negative geotaxis (B) and air-righting reflex (C) were delayed with pretreatment of the VN at P1 with CB1R agonist WIN55 and CB1R agonist AM251.

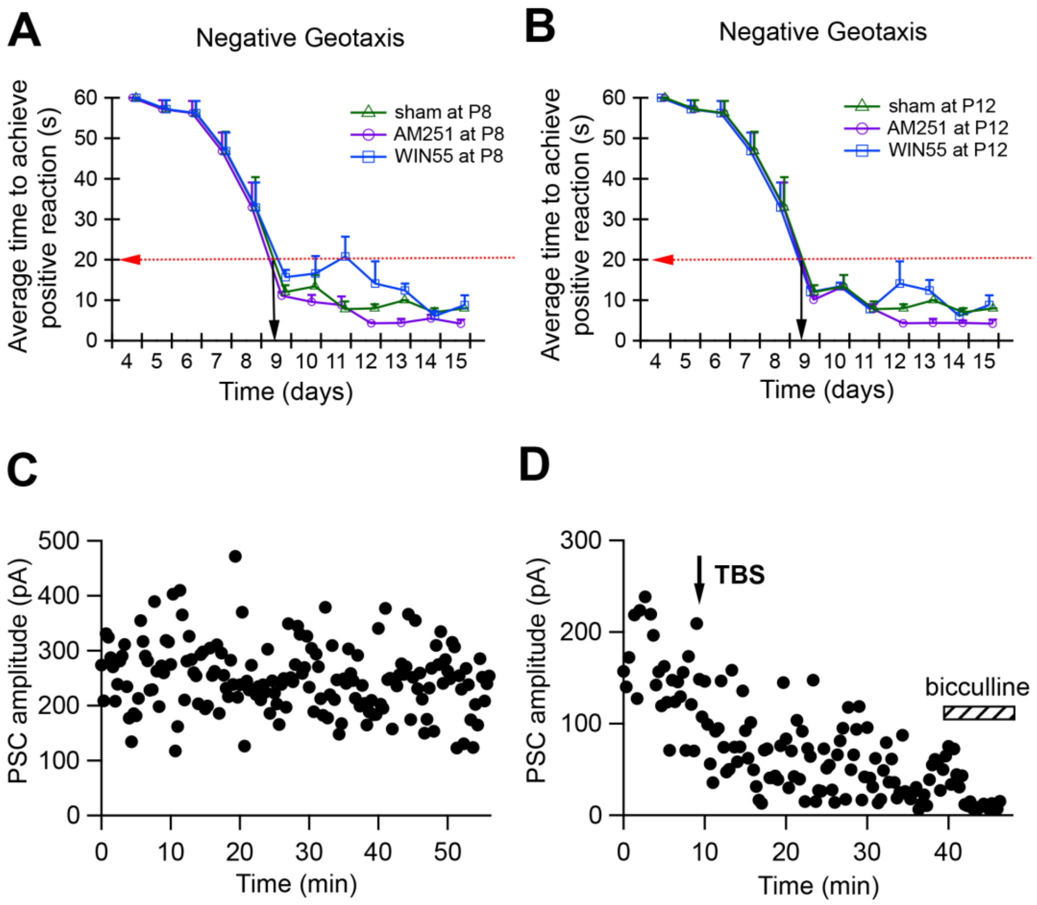

Fig. S3. Exposure to WIN55-loaded Elvax at P8 and P12 and representative PSC$_{GABA}$ responses, related to Figure 1. (A,B) Treatment with WIN55 or AM251 at P8 or P12 had no effect on the expression of negative geotaxis from nose-down to nose-up position (p=1). Three animal groups were tested: sham-control rats, rats pre-treated with AM251 or WIN55 at P8 or P12 (n=8 rats per group). The red dotted line shows the threshold time for defining positive reflex (within 20 s). (C) Representative baseline PSC$_{GABA}$ responses of a MVN neuron. (D) Representative tracing of a MVN neuron showing LTD of PSC$_{GABA}$ amplitude after TBS. PSC was blocked after bath addition of GABA$_A$ receptor antagonist bicculline, confirming the GABAergic identity of the recorded PSC. Mean ± SEM are shown. Two-way ANOVA for behaviral tests in (A), (B).

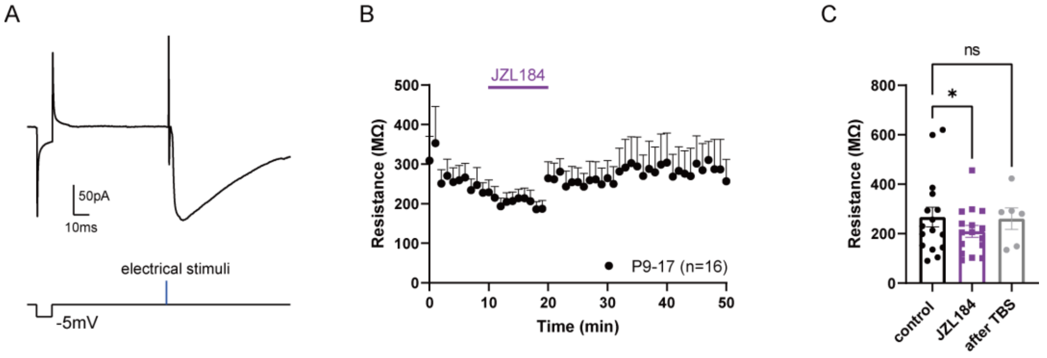

Fig.S4. Input resistance decreased with JZL184, related to Figure 1. (A) Current traces recorded during voltage pulses (−5mV). (B) Time course of normalized membrane resistance. (C) Quantification of membrane resistance.

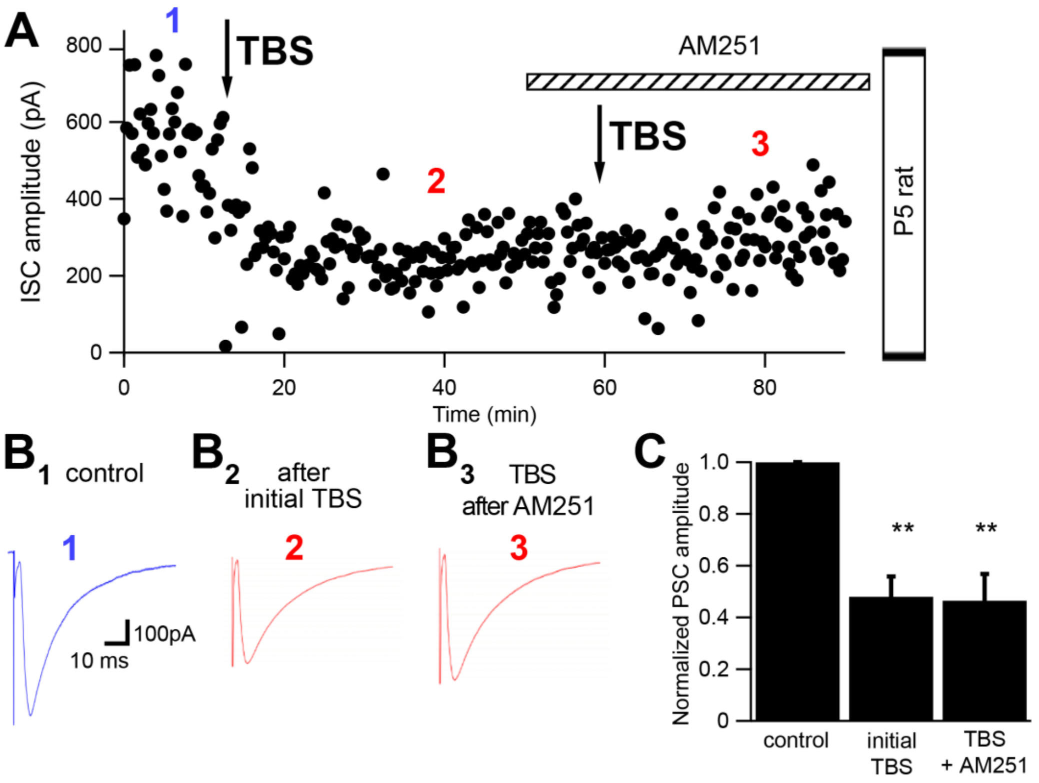

Fig. S5. LTD$_{GABA}$ response to a second TBS by bath addition of AM251, related to Figure 2. (A) A P5-8 rat VN neuron showing LTD response with an initial LTD did not show further response to a second TBS after bath addition of AM251. (B) Representative tracing of a single response in the baseline recording period (B_1), at 20 min after an initial TBS (B_2) and at 20 min after the second TBS with bath addition of AM251 (B_3). (C) Bar chart showing averaged normalized amplitude of PSC$_{GABA}$ in the baseline (0 – 10 min), after intial TBS (10 – 50 min), and after 2nd TBS with bath addition of AM251 (60 – 90 min). Mean ± SEM are shown. ** p<0.01, One-way ANOVA for comaprison of PSC$_{GABA}$ amplitudes in (C).

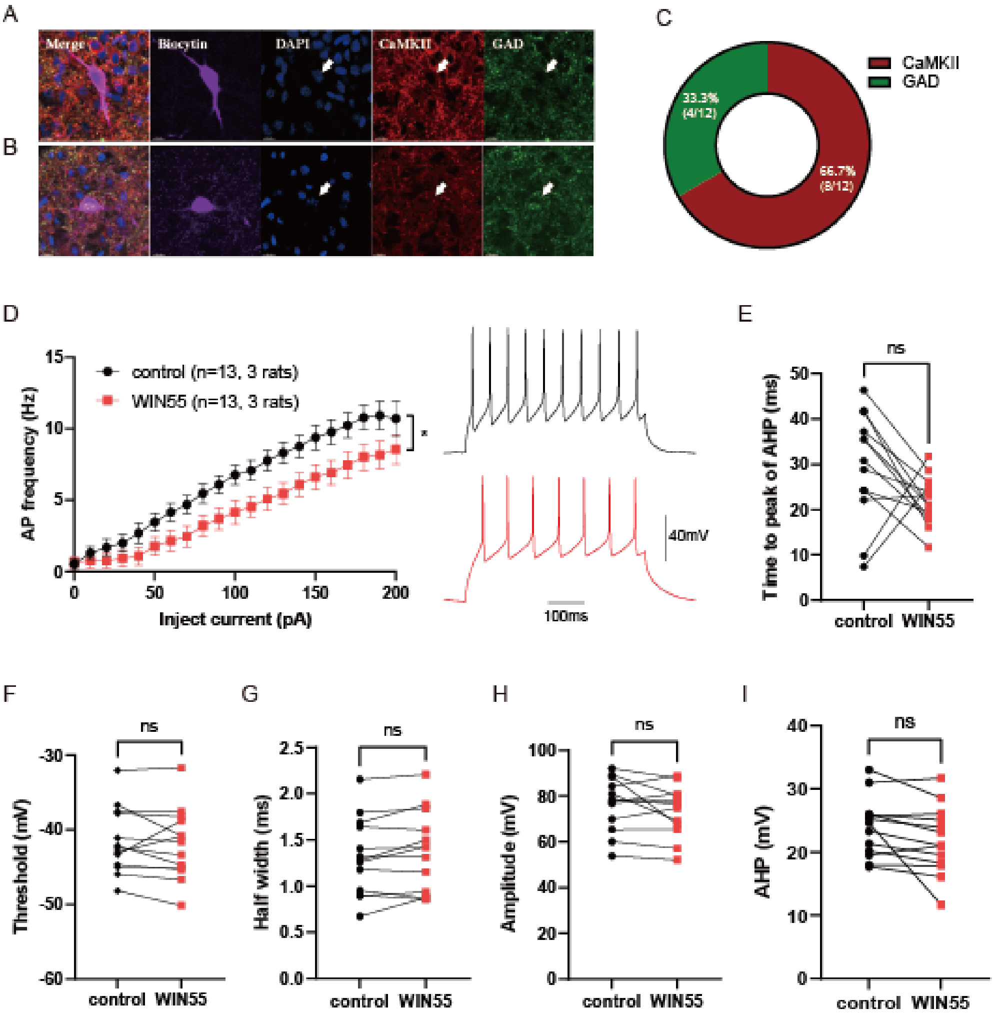

Fig. S6. Intrinsic excitability of VN neurons, related to Figure 2. (A) Immunostaining images of neurons co-labeled with CaMKII. (B) Co-staining images of neurons with GAD. (C) Proportions of different neuron types identified through co-staining. (D) Frequency-current relationship and representative spike trains of VN neurons from P9-17 rats, recorded in the absence (control, black) and presence (WIN55, red) of WIN55. The right panel displays the action potential firing pattern in response to a 150 pA current injection. (E-I) Summary of results showing comparable values for the time to peak of the AHP, action potential threshold, half-width, amplitude, and AHP duration, both with and without WIN55.

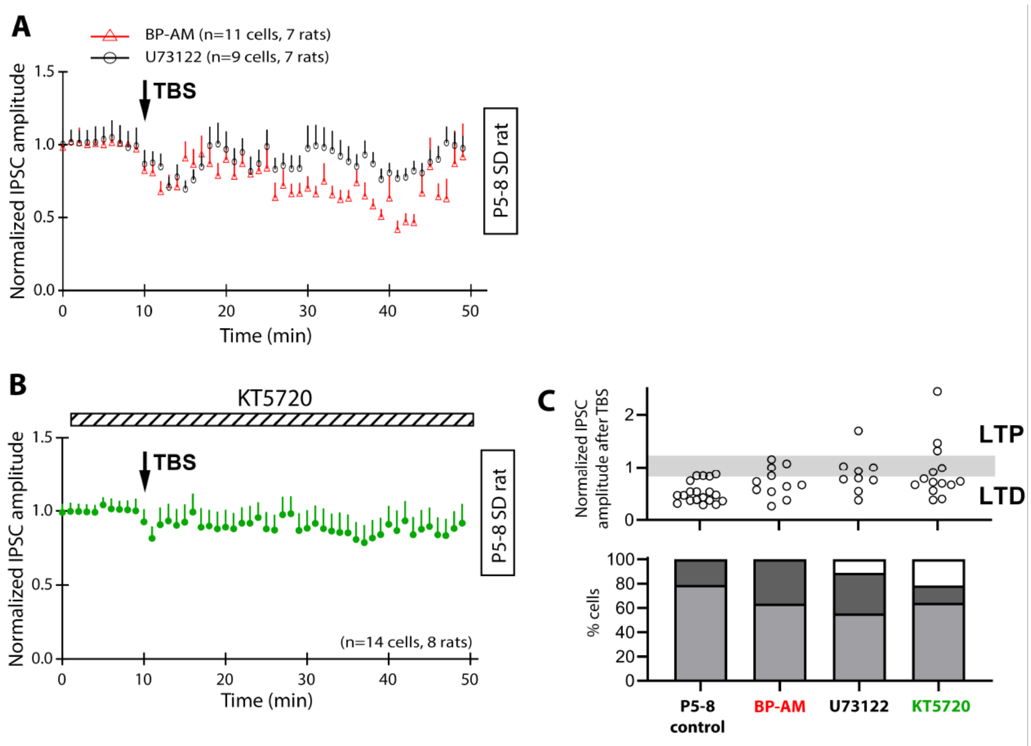

Fig. S7. Canonical PLC-, PKA-, and Ca^{2+}-dependent intracellular pathways are utilized by MVN neurons to effect eCB mediated LTD_{GABA}, related to Figure 3. (A) Average response of MVN neurons in P5-8 rat to TBS after bath addtion of BP-AM (a calcium chelator, red triangles). The occurrence of LTD decreased 64% comapred to 79% in age-matched controls (see panel C). In a separate preparation of P5-8 rat MVN neurons, bath addition of U73122 (a PLC blocker, black circles) decreased occurrence of LTD to 56% (see panel C). (B) Bath addition of KT5720 (a PKA blocker) to P5-8 rat MVN neurons decreased the occurrence of LTD to 64%. (C) Bar chart showing percentage of cells with LTD (light grey), no change (dark grey) or LTP (white). Mean ± SEM are shown in panels (A) and (B).

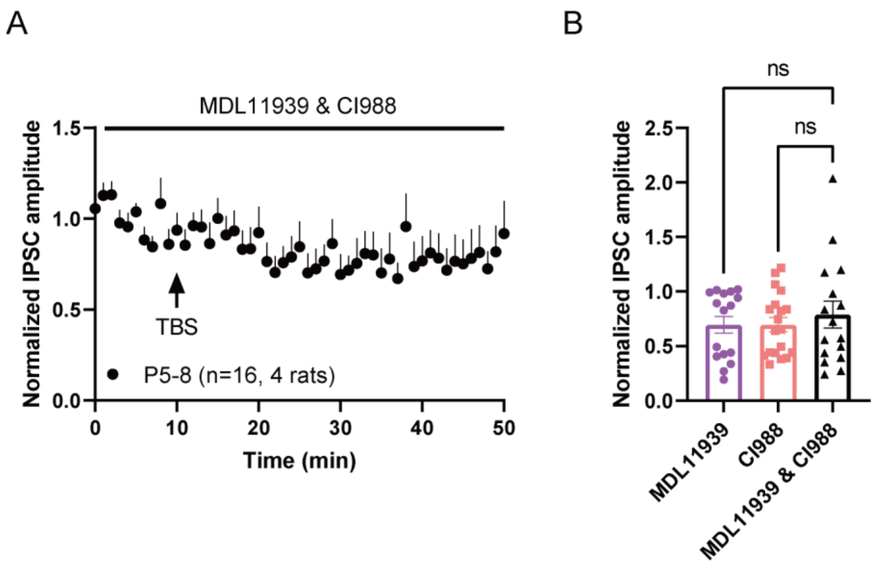

Fig.S8. Both MDL11939 and CI988 could not abolish LTD, related to Figure 4. (A) Group data (n= 16, 4 mice) of LTD induction with MDL11939 and CI988. (B) Summary of LTD in last 10 minutes with MDL11939, CI988 and both two antagonists.

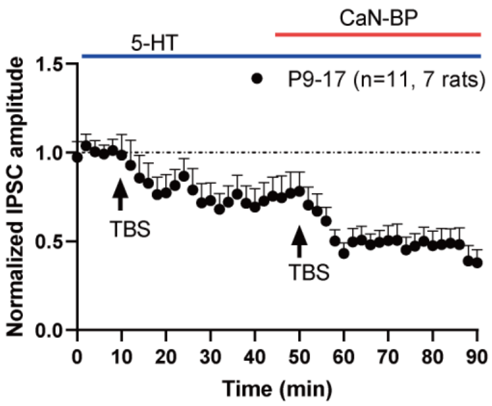

Fig.S9. MVN neurons in the presence of 5-HT that responded to an initial TBS with LTD_{GABA} could respond to a second TBS with further LTD_{GABA} after bath addition of CaN-BP in P9-17 brain slices, related to Figure 4.

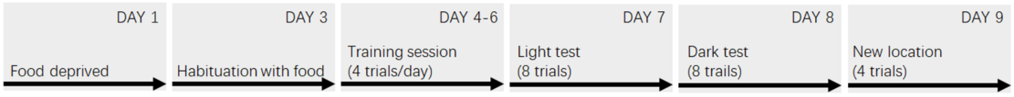

Fig S10. Schematics of dead reckoning test, related to Figure 6.

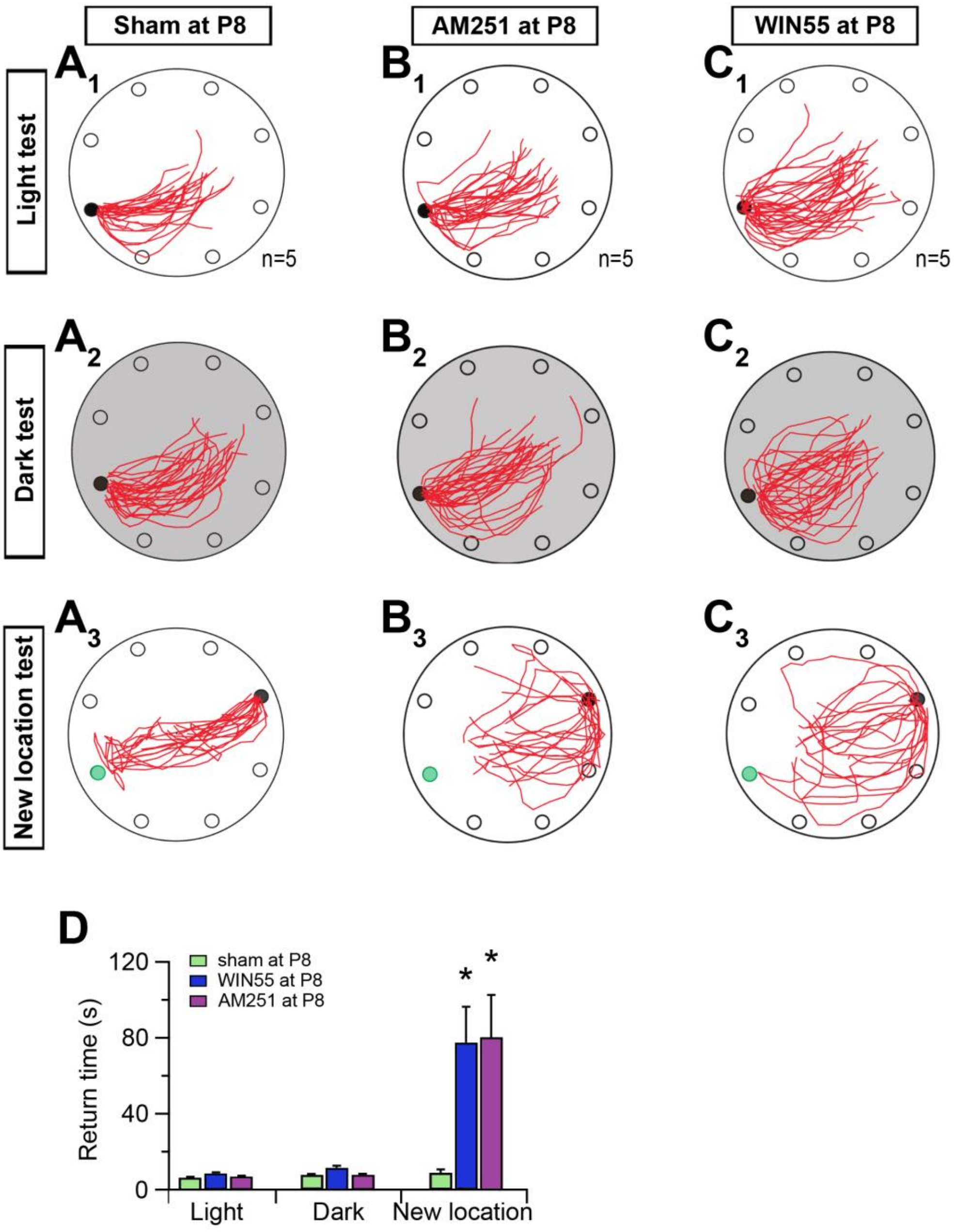

Fig. S11. Homeward paths of P60 rats with Elvax implantation at P8 in (A) light probe, (B) dark probe and (C) new home location probe in dead recokoning test, related to Figure 6. Filled black circles represent the location of the home base; green circles in panel (C) represent the location of the old home base. (D) Bar charts showing the average return time for rats in each treatment group. Mean ± SEM are shown. * $p<0.05$. One-way ANOVA for comparison of return time in (D).

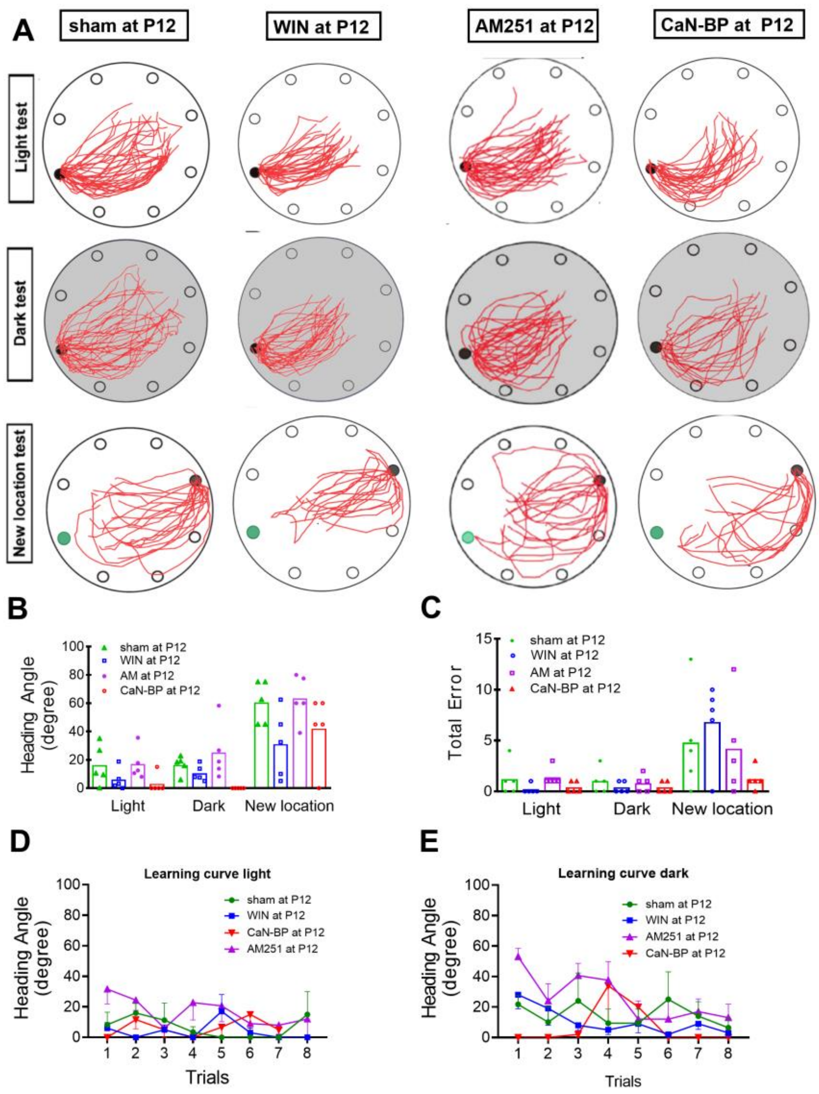

Fig S12. Perturbation of eCB signaling in the neonatal VN at P12 leads to no long-lasting deficits in vestibular-dependent navigation, related to Figure 6. (A-D) Excursion paths of adult rats pretreated at P12 with sham implantation, AM251, WIN55, or CaN-BP in the light, dark and new location test for spatial reckoning. Red lines represent the trajectories of homeward paths (light/dark test: 8 trails/rat are superimposed; new location: 4 trails/rat are superimposed). Filled black circle on the edge of each round table surface represents the location of the home base; green circles in bottom panels represent the location of the old home base. Histograms showing the average heading angle (E1), time spent in the quadrant containing food (E2), errors made in locating the homebase (E3), and learning curve for rats pretreated with AM251 (n=6), WIN55 (n=6), CaN-BP (n=5), or sham operated (n=5) at P12 (E4). Means ±SEM are shown. *P <0.05, **P <0.01. Two-way ANOVA for comparison of behavioral test performance in (E).

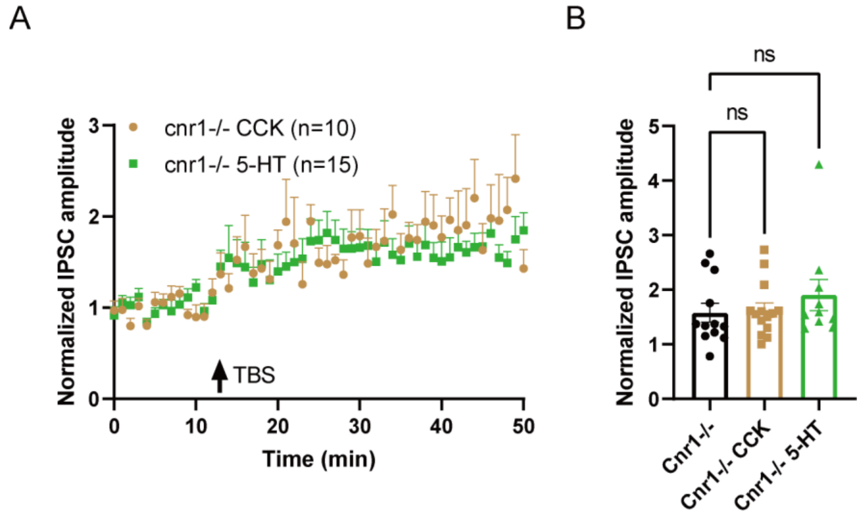

Fig.S13. Plasticity of P9-17 Cnr1$^{-/-}$ mice showed no change with CCK and 5-HT in bath, related to Figure 4. (A) Cnr1$^{-/-}$ mice expressed LTP with CCK and 5-HT. (B) Last ten minutes of normalized IPSC after TBS induction.

Methods S1: Supplementary Methods

Animals

Sprague Dawley rats (Charles River Lab), C57Bl6/J mice (Jackson Laboratory), vesicular GABA transporter (VGAT)-Venus transgenic mice (19) (a gift from Professor Y Yanagawa, Gunma University Graduate School of Medicine), and CB1R knockout ($Cnr1^{-/-}$) mice (Shanghai Model Organisms Center Incorporated) were used. For experiments conducted on adults, only male rats were used. Early postnatal animals were randomly picked for electrophysiological and behavioral experiments as the sex of rodents prior to weaning is not explicit. Procedures were approved either by The University of Hong Kong Committee on the Use of Live Animals in Teaching and Research or by Beihang University Ethics Review Board.

Elvax Slice preparation

200 μl of 10 mM CB1 receptor agonist WIN55 and/or 10 mM CB1 receptor antagonist AM251 solution in dimethylsulfoxide (DMSO, 4%, Sigma) were mixed and snap frozen with a 10% (w/v) Elvax solution in dichloromethane as described previously. Solidified slices were kept at -20 C to allow evaporation of dichloromethane. Slices cut to final dimensions (1 mm x 1 mm, 200 μm thickness) prior to implantation to the 4th ventricle via the foramen magnum.

Elvax slices (1 mm x 1 mm, 200 μm thickness) containing 10 mM ^{3}H-WIN55 and/or ^{3}H-AM251 were prepared and incubated in 0.5 ml phosphate buffered saline (PBS) at 37 °C to estimate the extent and time course of drug release. (Supplementary Fig. S1A). Penetration depth of drugs into the VN was determined by assaying radioactivity of horizontal brainstem slices at P21 after implantation of radioactive slice at P1. Radioactivity above background was not detectable beyond 600 μm from the medullary surface (Supplementary Fig. S1B). This confirmed that diffusion of the drugs remained within the MVN which had a dorsoventral depth of ≥700 μm measured from the medullary surface throughout P0 – 21.

Behavioral Tests

Negative geotaxis reflex, conducted inside a dark box equipped with an infrared video recorder, was tested in rats for 3 times per day from P4 to P15 as previously described (4, 6). The age of the rats acquiring the reflex was defined as that at which 90% of the

rats showed a mature response, i.e. re-oriented from nose-down to nose-up position within 20 s by P9.

Air righting test for vestibular-dependent graviceptive righting without interference from tactile and visual influences was performed on rats 3 times per day from P14 to P21. Videos of righting during free fall from a supine to a prone position were recorded 1,000 frames per second (RX10II, Sony) and analyzed offline. Trials in which rats pitched along the anterior-posterior axis were excluded. Percentage righting was calculated by number of successful rightings / total number of trials. The age at which rats acquired the reflex was defined as the age where percentage righting reached 80%.

Dead reckoning test for spatial cognition was conduction on P60 rats implanted with drug-loaded Elvax slices at P1, P8 or P12, and sham operated rats. Rats were fasted for 12 hr prior to test sessions. Only one food pellet (1 g, Supreme Mini-Treats, Bio-Serv) was provided during each test trial. Rats foraged for the food pellet placed randomly around the middle of the circular arena before returning to their home cage in 1 of the 8 possible locations around the arena. Stationary visual cues were provided during the training sessions and light probe tests. In the dark probe test, the lights were switched off and the arena was surrounded completely by a ceiling-to-floor black curtain. The new home location test was conducted in light with the home base moved to the hole diametrically opposite to its original home base. Eight trials were done in each of the light or dark probe tests and 4 in the new location test spread over 3 consecutive days (Supplementary Fig. S10). Heading angle, time in the quadrant with food, errors the rats made in return path, and the training time needed before rats learnt the task were measured from recorded video footage.

Brain Slice Preparation

The brain was removed from deeply anesthetized (5% isoflurane in 250 cm^3/min O_2) rodents, coronal brainstem sections (300 μm thickness) containing the MVN was cut under ice-cold aCSF using a vibratome (MA752, Campden Instrument). Slices were incubation in aCSF for 1 hr at 33 °C, and then kept at room temperature in aerated aCSF until use. During recording, the slices were perfused with aerated 32 °C (Warner Instrument Company) aCSF at a rate of 1.5 ml/min. Soma of neurons were visually identified by a 40x water immersion objective.

Drugs used for Electrophysiology

Drugs used are listed in Table S1. Drugs were dissolved at the maximum concentration and stored as aliquots at -80 ºC. Fresh aliquots were diluted into artificial cerebrospinal fluid (aCSF, composition in mM: 120 NaCl, 2.0 KCl, 1.2 $MgCl_2$, 2.5 $CaCl_2$, 1.2 KH_2PO_4, 11 glucose, and 26 $NaHCO_3$, pH 7.3, 285-295 mOsm, aerated with 95% O_2 and 5% CO_2) prior to use.

Whole-cell Patch-clamp Recording

Borosilicate glass pipettes (4-6 MΩ) filled with internal solution for voltage clamp containing (in mM): 140 KCl, 2 $MgCl_2$, 2 Na_2ATP, 1 ethylene glycol-bis (b-aminoethyl ether)-N,N,N',N'-tetra-acetic acid (EGTA), and 10 N-2-hydroxyethylpiperazine-N'-2-ethanesulphonic acid (HEPES) (adjusted to pH 7.3, 285-295 mOsm) were used. KCl-based internal solution was used to record evoked GABAergic postsynaptic currents ($ePSC_{GABA}$). For current-clamp recordings, electrodes were filled with an internal solution containing the following (in mM): 134 K-gluconate, 6 KCl, 10 HEPES, 4 NaCl, 7 K2-phosphocreatine, 0.3 NaGTP, and 4 Mg-ATP (pH 7.3 adjusted with KOH). No series resistance compensation was applied but the cell was discarded if the access resistance changed significantly (>25%) during the course of recording. Cell recording was discarded if the leaking current was >100 pA.

For perforated patch-clamp recording, gramicidin (Sigma-Aldrich) was added to the internal pipette solution to a final concentration of 5 µg/ml. Recordings started when the access resistance stabilized between 25–50 MΩ (29, 30). At least 5 eIPSCs at each holding voltage (-120 mV to 0 mV in 30 mV steps) were used to generate the current-voltage (I-V) graph.

Electrical stimuli (0.1 ms pulses delivered at 0.05 Hz) were delivered through bipolar tungsten stimulating electrodes (225 µm tip separation, Microprobes) placed at the vestibular afferents of the brainstem slice (4, 5). Response of neurons over the maximum recording duration was unchanged by repeated stimulation (Supplementary Fig. S2C). Theta burst stimulation (TBS) consisted of 4 epochs of 10 stimulus bursts delivered at 5 Hz each containing four pulses at 100 Hz. After TBS, IPSCs were recorded for at least 40 min. Levels of LTD-GABA or LTP-GABA were evaluated by comparing the averaged IPSC amplitude for 10 min just before TBS and that at 40 min after TBS. All $ePSC_{GABA}$ were recorded at -70 mV in aCSF containing D-AP5 (50 µM), CNQX (10 µM), and strychinine (1 µM).

Paired pulse ratio (PPR) was calculated using the amplitudes of the second PSC_{GABA} divided by the amplitude of the first from 30–50 consecutive recordings of paired stimuli

applied at 100-ms intervals with 0.05 Hz frequency. The minimum stimulation intensity able to trigger a single $ePSC_{GABA}$ was in the range 100–200 μA.

All miniature PSC_{GABA} ($mPSC_{GABA}$) was recorded at -70 mV in aCSF containing D-AP5 and CNQX and tetrodotoxin (TTX; 1 μM) to block action potentials.

In current-clamp, the action potential was evoked by injecting a stepped hyperpolarizing current (from −100 to 200 pA, 500 ms).

Electrophysiology: Data Analysis

$mPSC_{GABA}$ was analyzed using Mini Analysis v.6 (Synaptosoft Incorporated). The threshold for detection was set at 2-fold of the baseline noise. $mPSC_{GABA}$ frequency was calculated from a continuous recording of 10 min. Inter-event interval (IEI) was used to estimate the frequency of $sPSC_{GABA}$. The 10% rise time (rise time) and 80% decay time (decay time) were measured from single events in which no other rising and decay phases were present.

After recording, $ePSC_{GABA}$ was analyzed off-line using Digidata-pClamp package (Axon Instruments). $ePSC_{GABA}$ amplitudes were normalized using the average amplitude of events from 0–10 min before TBS. $ePSC_{GABA}$ were recorded from 10 to 90 min after TBS. The natural variation in basal synaptic strength between all recorded cells was up to 20%.

The first action potential (AP) elicited was used to assess AP threshold, amplitude, afterhyperpolarization (AHP), time to peak of AHP and half width. The threshold was measured as the membrane potential wherein dV/dt > 10 mV/ms during the AP rise phase. The AP amplitude was calculated between threshold and AP peak. The AP half-width was defined as the duration of half-amplitude between the threshold and peak. AHP were quantified from the first elicited AP and defined as the differences between the value of the peak hyperpolarizing voltage deflection following the AP and the AP threshold, time to peak of AHP defined as time between the action potential threshold and AHP.

Immunofluorescence

To label the patched neurons, 0.1% biocytin solution was added in K-Glu based intracellular solution. Afterward, the slices with biocytin-filled neurons were fixed in paraformaldehyde (PFA, 4% in 0.1M phosphate buffer) and kept at 4 ºC overnight. Slices

were first incubated with 1% BSA and 1% Triton-100 in 0.01M PBS for 1 h to block nonspecific binding and render cell membranes better permeability. Slices were then incubated with primary antibody, a mouse monoclonal anti-GAD antibody (ABclonal, diluted at 1:1000) and a rabbit monoclonal anti-CAMKII antibody (ABclonal, diluted at 1:1000), in 1% BSA and 1% Triton-100 in 0.01M PBS for 24 h at 4 ºC. After thorough rinsing with 0.01M PBS, the slices were incubated with secondary antibodies Alexa Fluor 488 goat anti-mouse IgG (ABclonal, diluted at 1:1000 and used for labeling GAD), Alexa Fluor 561 goat anti-mouse IgG (ABclonal, diluted at 1:1000 and used for labeling CAMKII) and Alexa-fluor 647-conjugated streptavidin (ABclonal, diluted at 1:1000 and used for labeling biocytin) at 4 ºC overnight. All images of stained sections were taken using a Confocal Microscope (Lecia, Mannheim, Germany) with 10, 20, or 40 objectives.

Statistical Analysis

All data are presented as mean ± SEM. One-way ANOVA followed by Bonferroni's correction for multiple comparison was used to compare the average time for accomplishing positive responses in negative geotaxis and air righting tests, as well as performance indexes in the dead reckoning test. Student's paired t-test was used to compare the average changes in the frequency and amplitude of $mPSC_{GABA,}$ and PPR of evoked PSC_{GABA} before and after TBS and action potential properties. Chi-squared test followed by Fischer's Exact test with Bonferroni's correction for multiple comparison was used to compare the response profiles of cells after TBS. One-way ANOVA followed by Tukey's test for multiple comparison was used to compare the amplitude and frequency of $sPSC_{GABA}$ or $mPSC_{GABA}$ among different age groups. The Kolmogorov-Smirnov test was used to compare differences between two cumulative distributions of amplitude or IEI of $mPSC_{GABA}$ for a given cell. Differences with $p < 0.05$ were considered statistically significant.
